# Distant activation of Notch signaling induces stem cell niche assembly

**DOI:** 10.1371/journal.pgen.1009489

**Published:** 2021-03-29

**Authors:** Andriy S. Yatsenko, Halyna R. Shcherbata

**Affiliations:** Institute of Cell Biochemistry, Hannover Medical School, Hannover, Germany; College de France CNRS, FRANCE

## Abstract

Here we show that multiple modes of Notch signaling activation specify the complexity of spatial cellular interactions necessary for stem cell niche assembly. In particular, we studied the formation of the germline stem cell niche in *Drosophila* ovaries, which is a two-step process whereby terminal filaments are formed first. Then, terminal filaments signal to the adjacent cap cell precursors, resulting in Notch signaling activation, which is necessary for the lifelong acquisition of stem cell niche cell fate. The genetic data suggest that in order to initiate the process of stem cell niche assembly, Notch signaling is activated among non-equipotent cells via distant induction, where germline Delta is delivered to somatic cells located several diameters away via cellular projections generated by primordial germ cells. At the same time, to ensure the robustness of niche formation, terminal filament cell fate can also be induced by somatic Delta via *cis-* or *trans-*inhibition. This exemplifies a double security mechanism that guarantees that the germline stem cell niche is formed, since it is indispensable for the adjacent germline precursor cells to acquire and maintain stemness necessary for successful reproduction. These findings contribute to our understanding of the formation of stem cell niches in their natural environment, which is important for stem cell biology and regenerative medicine.

## Author summary

Adult organs often contain a stem cell niche that maintains stem cells necessary for the replenishment of different types of terminally differentiated cells that are continuously lost. This study reveals that various modes of Notch signaling activation induce the formation of the germline stem cell niche in *Drosophila*. We show for the first time that even among non-equipotent cells, Notch signaling can be *trans-*activated via distant induction mode, where the ligand Delta is delivered via cellular protrusions to the somatic stem cell niche precursors located several cell diameters away. Moreover, there is a second security mechanism controlled by the soma that additionally ensures that the stem cell niche is formed. In the stem cell niche precursors, Notch signaling can be locally inhibited by the somatic Delta. While Notch signaling *trans-*inhibition has been proposed via mathematical modelling, our findings show that a group of cells that have high Delta can be seen in a living organism, confirming that this mode of Notch signaling inhibition by *trans-*Delta exists *in vivo*. This work provides significant advances in the understanding of Notch signaling and the stem cell niche formation, which is important for the fields of stem cell biology and regenerative medicine.

## Introduction

**Fig 1:**
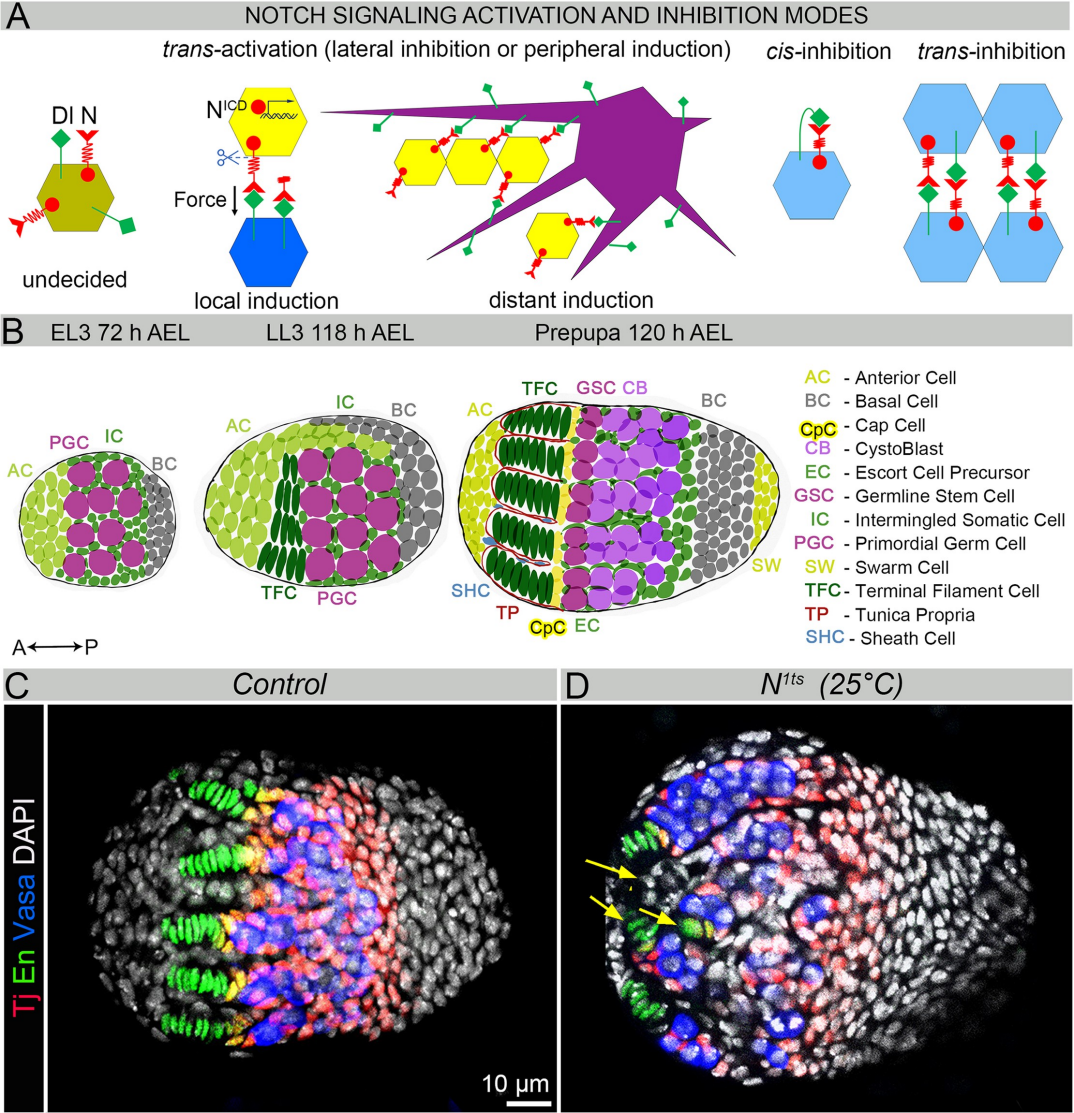
Ovarian morphogenesis is defective in Notch signaling mutants. (**A**) Models of Notch signaling activation and inhibition modes. During development, cells can express both Delta and Notch and be “undecided” with regards to the Notch signaling status (olive cell). However, typically, Notch signaling is actively induced via *trans-*interaction: a cell that expresses activated Delta ligand induces Notch signaling in the neighboring cells and represses their choice to become Delta-sending cells. This signaling is called “lateral inhibition” if among equipotent cells or “peripheral induction” if among non-equipotent cell populations. Peripheral induction can be divided into two different types: 1) local induction, when Notch signaling is activated in the cells whose membranes which are in direct contact with those of the Delta-expressing cell; and 2) distant induction, when Notch signaling is activated in the cells that are localized several cell diameters away with the help of cellular Delta-sending projections. As a result of the Notch-inductive mode, direct activation of the genes coding for factors establishing terminally differentiated cell fates is induced. In some cases, Notch signaling can be inhibited by the intrinsic or extrinsic interaction of the Delta ligand and the Notch receptor (*cis-* or *trans-*inhibition). Expressed in the same cell, the Delta ligand and Notch receptor can bind each other, leading to the repression of Notch signaling via *cis-*inhibition. Similarly, Delta that is not properly processed can bind the Notch receptor on the membrane of the adjacent cell, leading to the repression of Notch signaling via *trans-*inhibition. (**B**) Schematic of the ovary at different stages of *Drosophila* development. See the color-coded legend which is referencing different types of cells in the ovary. (**C-D**) Examples of wild type (**C**) and *Notch* mutant (*N*^*ts1*^ at 25°C during L3, **D**) prepupal ovaries. Note the abnormal organization of the GSC niche units in *N*^*ts1*^ mutants. TFCs are marked by En (green). ICs are marked with Tj (red). CpCs express both Tj and En (yellow). Germline is marked with Vasa (blue). DAPI marks cell nuclei (white). Arrows indicate disoriented and abnormally shaped TFs.

The Notch pathway is an evolutionarily conserved signaling pathway that presents a great assortment of complex behaviors in various developmental situations. During development, cells often express both the Delta ligand and Notch receptor; therefore, they are undecided with regards to Notch signaling status (Fig 1A). This indecisiveness can be resolved randomly over a period of time; for example, when one cell expresses more Notch or Delta due to transcriptional noise, then the adjacent cell accepts the opposite cell fate [1–3]. However, most of the time, in order to generate a stable Notch signaling pattern required for proper tissue organization, Notch signaling is activated via cell-to-cell interactions.

In particular, among a group of equivalent cells, Notch signaling activation induces mutually exclusive cell fates in the adjacent cells in a process called “lateral inhibition”. In this case, the membrane-localized Notch ligand (Delta or Serrate) binds to the Notch receptor on the membrane of the neighboring cell. With the help of mechanical forces, Delta-Notch interaction causes Notch receptor cleavage and translocation of its intracellular domain to the nucleus. There it serves as a transcriptional co-factor that activates expression of Notch-dependent genes, which include repressors of Delta ligand [4–6]. This typical short-range Notch activation via lateral inhibition is seen in multiple tissues of evolutionarily diverse species [7–11]. In addition, it has been shown that in the epithelial cells within imaginal discs, long-range Notch lateral inhibition exists, which is mediated by Delta-promoted planar filopodia. These dynamic filopodia transmit intermittent Delta-Notch signaling, which refines Notch signaling during mechanosensory bristle formation and even can promote tumorigenesis in the mesenchymal cells [12–15].

In contrast to lateral inhibition, which occurs between equipotent cells, Notch signaling is activated via “peripheral induction” between non-equipotent cells. In particular, here we distinguish two types of inductive Notch signaling: if non-equipotent, communicating cells are juxtaposed and Notch signaling is activated in those cells adjacent to the Delta-sending cell, it is called “local induction”; if non-equipotent, communicating cells are distant and a plane of Delta-sending cells signals to one or more layers of Delta-receiving cells, it is called “distant induction” (Fig 1A). Importantly, local induction is sharp, while distant induction could result in graded activation of Notch signaling. In all of the Notch signaling modes described above, Notch signaling activation occurs as a result of *trans-*interactional communication that happens between the cells, membranes of which are in direct contact or between detached cells with the help of cellular projections. In each mode, amounts of Notch and Delta presented on the interacting membranes are different; therefore, the efficiency of Notch signaling activation depends on cell-cell contact geometry [16].

In addition to *trans-*activating, Notch and Delta expressed on the same cell surface can form *cis-*inhibitory complexes, resulting in intrinsic Notch signaling repression [17–20]. Recently, a *trans-*inhibition mode has been proposed, wherein Delta just binds Notch at cell-to-cell junctional contacts, which, without mechanical pulling force, does not translate into Notch receptor cleavage and activation. This allows larger groups of neighboring cells to express high levels of Delta [21].

In all cases, acquisition of a certain Notch signaling status generates mutually exclusive signaling states between groups of cells that subsequently activate genes coding for factors establishing terminally differentiated cell fates [4,6,14,22].

To study the interplay of Notch signaling activation modes necessary for proper cell fate acquisition and tissue patterning *in vivo*, we focused on the process of *Drosophila* ovarian stem cell niche formation as a model system. In the *Drosophila* ovary, the stem cell niche size directly depends on the strength of Notch signaling: when Delta ligand is overexpressed, it induces enlarged, functional niches, regardless of whether Delta is overexpressed in the germline or soma [23,24]. These data imply that the stem cell niche morphogenesis could be differentially patterned depending on the strength and the source of Delta expression. However, it is undetermined what is the source of Delta and whether the germline communicates with the soma to confer Notch patterning necessary for proper stem cell niche formation.

The onset of *Drosophila* ovarian morphogenesis is marked by a merger of the primordial germline cells (PGCs) with the somatic gonad precursor cells, which occurs during early embryonic development (Fig 1B). This results in the formation of round, paired gonads in the abdominal region of the embryo, in which germline and somatic cells are intermingled. Both cell types divide mitotically and do not differentiate [25–28]. Only after the second instar larval stage [L2, 72h after egg laying (AEL)], based on their anteroposterior location within the embryo, somatic cells begin to differentiate into round-shaped apical (ACs) and basal cells (BCs), as well as squamous intermingled somatic cells (ICs) that envelop individual PGCs (Fig 1B). At the late third instar larval stage (LL3, 118h AEL), the ovary elongates, as a group of ACs called swarm cells (SCs) begins to migrate posteriorly, probably inducing mechanical forces that allow changes in the shape of the organ [29]. The differentiation of somatic cells progresses, resulting in the appearance of short cylindrical terminal filament cells (TFCs) that form separate terminal filament (TF) stalks (Fig 1B). At the base of each stalk, six ICs change their shape into ellipsoid and acquire a stem cell niche cell fate for life [26,30,31]. These cells are called cap cells (CpCs), and they generate signals necessary for germline stem cell maintenance. Therefore, PGCs that are in direct contact with CpCs become germline stem cells (GSCs), while other PGCs that are enveloped by escort cell precursors (ECs) will differentiate [23,24,32–37]. This concludes the assembly of the functional GSC niche unit, which consists of one TF, CpCs, antrerior ECs, and GSCs and is responsible for GSC self-renewal during the lifetime. Individual GSC niche units are separated by extracellular matrix deposited by a group of posteriorly migrating ACs called sheath cells (SHC), resulting in the formation of individual ovarioles.

Thus, the GSC niche formation is a sequential process in which the TFs are assembled first, followed by specification of CpCs, which act as the niche cells [31,33]. While it is known that Notch signaling activation is essential for the GSC niche cell fate [23,24,33,38,39], it is not clear whether it also plays a role in TF cell specification. To understand the role of Notch signaling in the stem cell niche assembly, we aimed: *i)* to identify the physiological sources of Delta that chronologically induce Notch signaling in the somatic cell precursors and *ii)* to distinguish via what modes Notch signaling is activated in the process of acquiring different cell fates by niche cells.

Here we found that the key components of the pathway, Delta and Notch, have dynamic expression patterns, which affects the spatiotemporal pattern of Notch activity. Our data show that the Notch signaling pathway controls both major steps of GSC niche formation (TF assembly and CpC specification), and its activation modes vary significantly depending on the cell type and the sources of the Delta ligand. First, acquisition of a certain Notch signaling status (ON or OFF) is required for proper TF assembly. Depending on the TFC precursor position with regards to the germline, it would have Notch signaling either activated by the germline Delta via *trans-*interaction or inhibited by the somatic Delta. Importantly, the germline delivers Delta across several cell diameters to the somatic precursor cells plausibly via PGC-generated projections, resulting in the long-range induction of Notch signaling. At the same time, to ensure robustness of ovarian organogenesis, TF cell fate can be also induced by the inhibition of Notch signaling in the somatic TF precursors. Since TF establishment is a pre-requisite for the stem cell niche formation, activating and inhibitory Delta interaction modes offer a double security mechanism which guarantees that these events occur. Later, the Delta ligand expressed by the posterior TF cell activates Notch signaling in the adjacent somatic cells and transforms them into CpCs, which concludes the two-step process of the stem cell niche formation. Notch-induced cell differentiation is accompanied by alteration in size, shape and position of TFCs and CpCs. In summary, our data show that in the process of germline stem cell niche assembly, multiple layers of Notch signaling pathway activation (ON/OFF, low/high) define the accuracy of cell fate choice.

## Results

### Notch signaling mutants have impaired GSC niche morphogenesis

To study the role of Notch signaling in coordination of stem cell assembly, we analyzed and compared developing ovaries of wild-type and Notch signaling mutants. Conveniently, in the developing ovary, all different cell types can be easily identified using specific markers. Upon differentiation, precursor cells start to express distinctive sets of proteins; for example, TFCs can be marked by expression of the transcription factor, Engrailed (En, Fig 1C), while ICs express another transcription factor, Traffic jam (Tj, Fig 1C), and CpCs express a combination of these two factors (En+Tj, Fig 1C, green+red = yellow). The PGCs can be distinguished based on the presence of proteins that are exclusive for the germline (*e*.*g*., Vasa, Fig 1C, blue). Using these multiple markers, we analyzed the organization of the prepupal ovaries, in which niche formation is already completed. We compared the GSC niches of controls and *Notch* signaling mutants, in particular, we studied *Notch* (*N*^*ts1*^) temperature-sensitive mutants. Due to lethality, these mutants were kept at the restrictive temperature (25°C) only during the timeframe of the niche formation (late L2—early prepupa). We found that stem cell niche morphogenesis was severely affected in *N*^*ts1*^ mutants—TFs were abnormally shaped, had irregular length and lost their anteroposterior orientation (Fig 1D, arrows). These data suggest that Notch signaling is involved in the process of germline stem cell niche assembly, the TF morphogenesis.

### In the developing ovary, the pattern of Notch signaling activation is dynamic

Since the dynamics of Notch signaling in the developing ovary have not been studied before, we analyzed the timing and spatial expression pattern of the key components of Notch signaling–the major Notch signaling ligand, Delta and its receptor, Notch, as well as a Notch activity reporter during different stages of ovarian morphogenesis. It is important to note that depending on the Notch signaling status, the expression pattern and subcellular localization of the Notch receptor and the Delta ligand change. When Notch signaling is activated, membrane-localized Notch interacts with the activated membrane Delta from the other cell (Fig 1A, blue and yellow cells). This generates a pulling force resulting in Notch cleavage and translocation of its intracellular domain to the nucleus [40]. Since endocytosis is essential for Delta activation, activated Delta can be distinguished by its vesicular localization [41]. Nuclear Notch is difficult to detect with antibodies; therefore, Notch signaling activation is depicted as membrane Notch disappearance [42]. However, absence of the signal can also result from the inhibition of Notch expression; therefore, it is more reliable to analyze Notch signaling activation using Notch signaling reporters. At the same time, co-analysis of Notch and Delta expression patterns can be helpful to analyze other Notch signaling modes, such as when Notch signaling is inhibited [18,21]. In these cases, high levels of non-activated Delta are present at the cell membranes. This Delta can bind the Notch receptor in *cis-* or *trans-*, but cannot cause its cleavage and activation, leading to Notch signaling inhibition (Fig 1A, light blue cells). Therefore, high Delta and low Notch are co-present on the membranes of the cells in which Notch signaling is inhibited; however, it is impossible to distinguish between extrinsic Delta*-trans* and intrinsic Delta*-cis* inhibition. In addition, cells that express relatively high levels of Notch and Delta can be still undecided with respect to their Notch signaling status (Fig 1A, olive cell).

**Fig 2:**
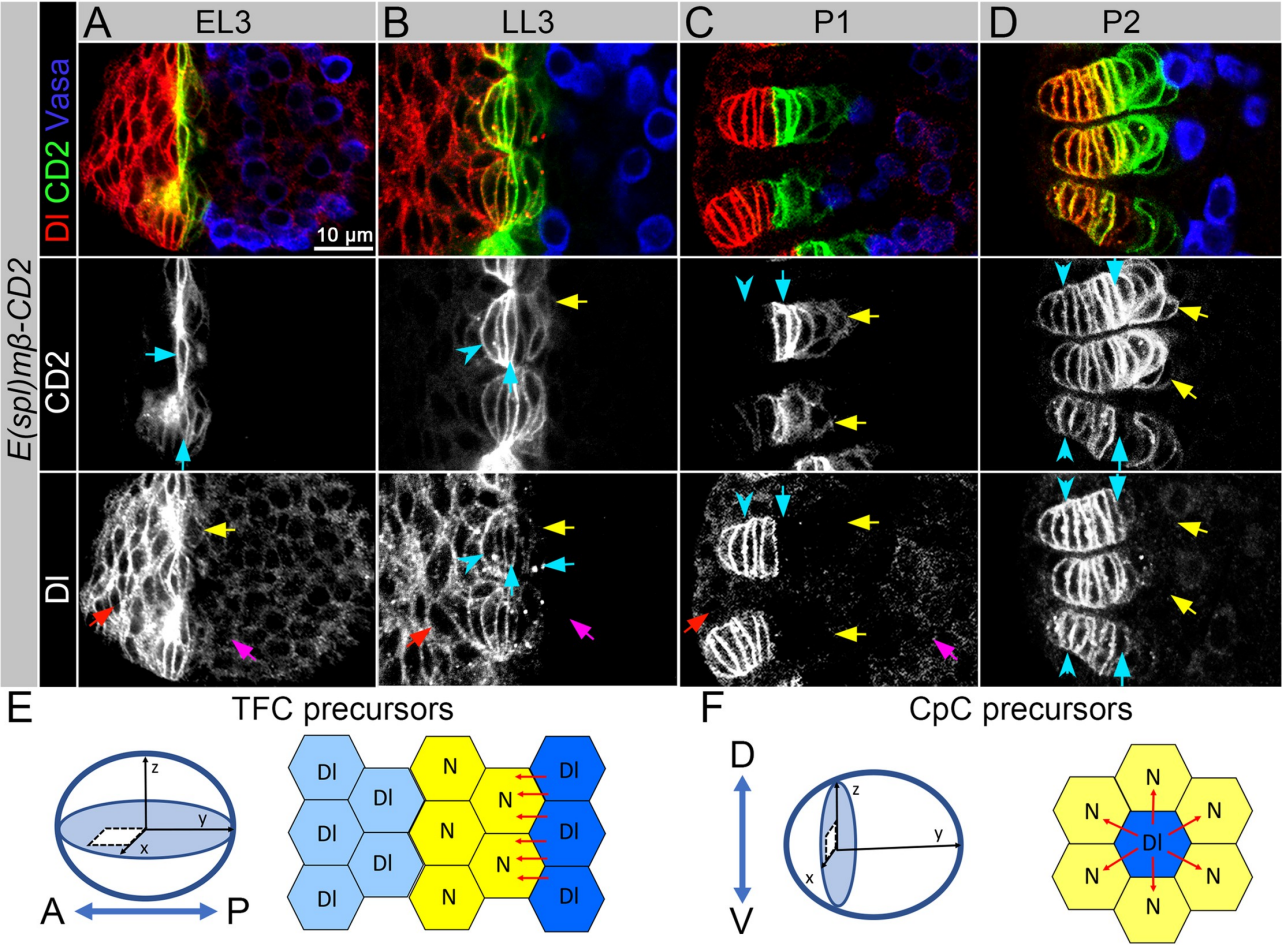
Patterning of Notch signaling at different stages of ovarian morphogenesis. (**A-D**) Co-expression of the Notch activity reporter (*E(Spl)mß-CD2*, green) and the Delta protein (red) in the developing ovaries. Vasa (blue) marks the germline. Single channels for the Notch activity reporter (CD2) and Delta (Dl) are shown below. At EL3 stage, Notch activity is first detected in the early TFCs (**A,** cyan arrows). At LL3 and prepupa stages (P1 and P2), lower levels of Notch signaling activity are detected in CpCs (**B** and **C**, yellow arrows) and higher levels in the posterior TFCs (cyan arrow). Note the absence of Notch signaling activity in the anterior TFCs (cyan arrowheads in LL3 and P1). After TFCs and CpCs are specialized, a dynamic Notch signaling activity pattern is observed in these cells (cyan arrow and arrowheads, P2). During all stages, Delta is expressed in ACs (red arrows) and in the germline ICs (magenta arrows); however, its expression levels notably decrease in these cells as organogenesis progresses. In TFCs, Delta expression shows complex dynamics, first, all TFC precursors have high levels of Delta (EL3), as TFCs are organized into individual stalks, Delta levels remain high in the anterior TFCs (cyan arrowheads) and are reduced (LL3) and then, absent (P1 and P2) in the posterior TFCs (cyan arrows). Note that some of the posterior TFCs have vesicle-internalized Delta (horizontal cyan arrow), which is important for Notch signaling activation and induction of the stem cell niche cell fate in CpC precursors (**B**, yellow arrows). See also the images of the expression patterns of different Notch activity reporters (S2 Fig). **(E-F**) GSC niche development is a two-step process, which includes TF formation and CpC specification. Schematics showing positioning and the proposed modes of Notch patterning induction within the developing ovary in TFC precursors (**E**) and CpC precursors (**F),** based on [31]. Anterior ↔ Posterior (A↔P). Dorsal ↔ Ventral (D↔V).

Firstly, to detect active Notch signaling, we used a Notch signaling reporter in which the sequence encoding rat CD2 protein is inserted downstream of the Enhancer of Split [*E(spl)mß*] promoter, which is activated by Notch signaling [43]. We were intrigued to note that the activation of Notch signaling coincides with the onset of GSC niche morphogenesis. In particular, at the early third instar larval stage (EL3) a small group of ACs adjacent to the germline start to express the Notch signaling reporter (Fig 2A, cyan arrows). Then, at later stages, CpCs adjacent to TFs also have active Notch signaling (Fig 2B–2D, yellow arrows). This suggests that in the stem cell niche precursor cells, Notch signaling plays a role in the terminal cell fate acquisition by both cell types comprising the stem cell niche unit (TFCs and CpCs).

Secondly, we analyzed Delta and Notch protein localization and found that both proteins have highly dynamic spatiotemporal expression patterns (Figs 2 and S1A–S1C). In particular, the Delta ligand is expressed in both the germline and the soma (Fig 2A–2C, lower panel). In the germline, higher levels of Delta are observed at earlier stages, and as ovarian morphogenesis progresses, Delta levels decrease (magenta arrows). We confirmed the specificity of Delta protein expression in PGCs by analysis of mutant ovaries with germline-specific Delta downregulation (S1D and S1E Fig).

Similarly, in the ovarian soma, the Delta expression pattern is dynamic, and Delta levels in general decrease at later stages of development. At EL3 stage, all ACs, including precursors of TFCs, express both Delta ligand and Notch receptor (compare lower panels in Figs 2A and S1A–S1C). Later, the expression of the Notch activity reporter indicates Notch signaling activation in TFCs. However, we noticed that not all TFCs turned “on” Notch signaling (Fig 2B, anterior and posterior TFCs, cyan arrowheads and cyan arrows, respectively). In particular, anterior TFCs express high levels of Delta and do not express the Notch activity reporter, suggesting that Notch signaling could turned “off” in these cells by the somatic Delta. At the same time, posterior TFCs adjacent to the germline lose membrane Delta and Notch and express the Notch activity reporter, suggesting that Notch could turned “on” in these cells by the germline-originated Delta.

The analysis of expression patterns of the Notch receptor and Delta ligand in CpC precursors shows that at early stages of development (EL3), ICs express both proteins (Figs 2A and S1A, yellow arrows), indicating that these cells are in an undecided state. Later, at LL3 and Prepupa, the presence of Delta and Notch at the membranes of ICs is strongly reduced (Figs 2B, 2C, S1B and S1C, yellow arrows). This accords with the onset of Notch signaling activation in a group of ICs adjacent to the TFs, which actually acquire the CpC fate (Figs 2B, 2C and S1A, yellow arrows). Notably, the efficiency of Notch signaling activation in CpCs, as detected by the expression levels of the Notch activity reporter, is lower than in TFCs, indicating that the intensity of Notch signaling is different in these two types of stem cell niche cells.

In summary, the expression pattern analysis demonstrates that the dynamics of Notch signaling in the developing niche cells are spatiotemporally complex and depend on the cell differentiation state. In particular, it is interesting that cells have to switch from the bivalent (Notch and Delta co-expression) to the decided (Notch “off” or Notch “on” and Notch “high” or Notch “low”) state in the course of their differentiation. Next, we wanted to understand how Notch signaling activation is accomplished in these cells during ovarian morphogenesis.

### Stem cell niche precursors acquire active Notch signaling status via different mechanisms

TFCs and CpCs derive from different types of somatic precursor cells: TFCs from ACs that are anteriorly located in the ovary; and CpCs from ICs that are medially located and intermingled with PGCs [29,32,33,44]. Upon differentiation, these cells adopt quite distinct shapes: cylindrical TFCs that arrange in the shape of a string (Fig 2E) and ellipsoid CpCs that unite in a shape resembling a flower with six petals (Fig 2F). They also have very different Notch/Delta expression patterns; however, both cell types acquire a certain Notch signaling status as they undergo terminal differentiation (Fig 2C, CD2, cyan and yellow arrows). Since TFCs and CpCs are spatially organized in a different manner and temporally induced to differentiate at different stages, we wanted to understand the sources of Delta signal that specifically control their organization and induce their active Notch status.

We analyzed Notch activity and Delta protein expression in a single TF in greater detail and found that Notch signaling displays a differential activity pattern as visualized by the fluorescent intensity of the Notch activity reporters (S2 Fig). In particular, the TFCs positioned next to the germline showed Notch activation, while cells at the anterior half of the TF showed no Notch activity (S2 Fig). Thus, the response to Delta observed here suggests that for the posterior TFC specification, Notch signaling activation occurs via an inductive mode, in which Delta signal originating from the germline activates Notch signaling via *trans-*interaction in the adjacent TFC precursors via distant induction (as schematically depicted in Fig 1A).

At the same time, anterior TFCs are positioned >4 cells away from the germline, which makes them less accessible for *trans-*interactions. Unlike the posterior TFCs, instead of the Notch signaling activity reporter, they express high Delta (Fig 2B and 2C, cyan arrowheads). Thus, the analysis of Notch signaling activation in the TFCs shows that despite the fact that TF cells originate from the same somatic precursor cells (ACs), look identical, and are similarly organized into a filament, they are quite different in terms of Notch signaling activity. We propose that anterior TFCs have Notch signaling inhibited possibly via intrinsic or extrinsic Delta-Notch inhibition, while posterior TFCs have Notch signaling induced via *trans-*activation as a response to Delta produced by the germline (Fig 2E).

Notably, the most posterior TFC (S2A Fig) has weaker Notch signaling activity due to the fact that the status of this cell (with regards to Notch signaling) is reprogrammed, which happens after the TF is formed and in response to steroid signaling [31]. Actually, this Delta-sending TFC serves as a source of the Delta ligand, which via peripheral induction induces Notch signaling in the adjacent niche cell precursors to convert them into CpCs and to ensure the formation of the stem cell niche (Fig 2F).

Importantly, Notch signaling activity continues to be very dynamic and changes as ovarian organogenesis progresses. After the process of stem cell niche differentiation is finalized, all TFCs and CpCs have Notch signaling activated (Fig 2D), while in the adult germarium, only few TFCs and CpCs have Notch signaling “on” (S3 Fig). During adulthood, CpCs co-express Notch and Delta and according to the rules of lateral inhibition, if at least one cell shifts the balance to express more Notch or Delta, it immediately will initiate the opposite (Delta or Notch) cell status in the adjacent neighbor [7,19,22,45]. Thus, CpCs have a stochastic Notch activity pattern (S3 Fig), suggesting that they *per se* can be responsible for the maintenance of Notch signaling activity in the adult GSC niche.

### Notch signaling plays a role in TF formation

The formation of individual TF stacks precedes CpC specification; however, the role of Notch signaling in TF morphogenesis has not been studied before. TF formation is induced by the rearrangements of anterior cells adjacent to PGCs, which coincides with an acquisition of Notch active status by these cells (as shown above). TF precursor cells cease their division, change shape from spherical to ellipsoid to cylindrical, and increase cell adhesiveness. This attracts the neighboring cells and, in a wave-like fashion, induces cell intercalation into TF stacks [26,44,46–49]. Since the expression analyses demonstrate that Notch signaling is differentially patterned in TF precursors, we hypothesized that the Notch pathway controls the formation of individual TF stacks, which is the first step of GSC niche morphogenesis.

**Fig 3:**
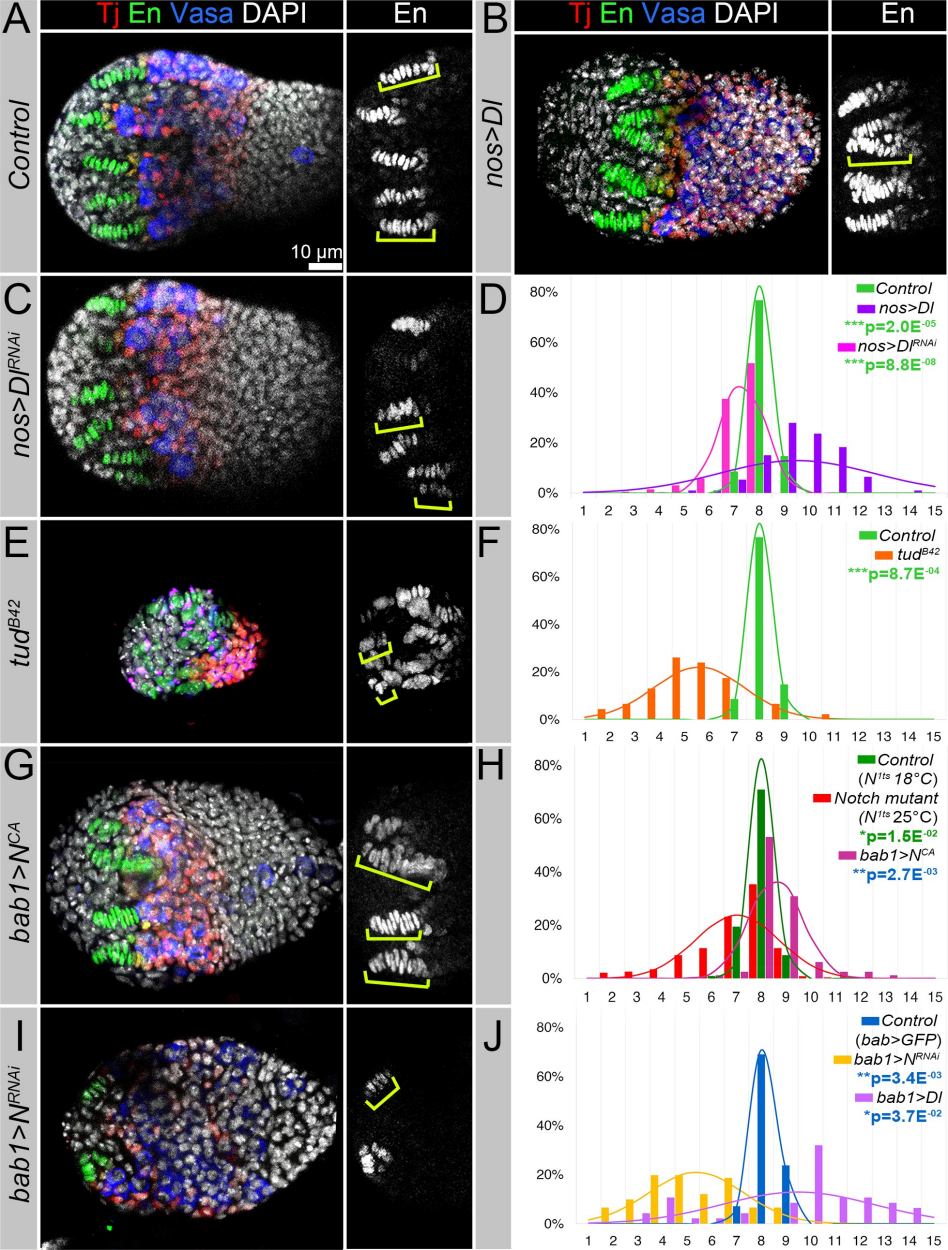
Germline Delta plays an instructive role, but it is not essential for TF assembly. (**A-D**) Upregulation of Delta in the germline (*nos>Dl*, **B**) increases, while downregulation of Delta in the germline (*nos>Dl*^*RNAi*^, **C**) decreases the number of TFCs/TF in comparison to control (*OregonR/w*^*1118*^, **A**). (**D**) The graph shows the observed frequency (histogram) and the probability (curve) of the TFC number per TF for mutants with the germline-specific up- and downregulation of Delta. See also S1A Table. (**E-F**) In the ovaries of *tudor* mutants that lack germline cells (*tud*^*B42*^, **E**) the number of TFCs/TF is decreased (**F**) due to the fact that Notch signaling was not activated in TFC precursors by the germline Delta (no germline induction). See also S1A Table. (**G-H**) The presence of the Notch receptor is important for the activation of Notch signaling in TFC precursors. Notch deficiency (*N*^*ts1*^, 25°C, **H**) affects TF formation, since the germline Delta cannot induce Notch signaling in the Notch receptor-deficient cells (no *trans-*activation). This results in the appearance of TF with the significantly reduced TFC numbers when compared to control (*N*^*ts1*^, 18°C, **H**). Also, forced expression of the constitutively active Notch receptor in TFC precursors results the appearance of TFs containing more TFCs (*bab1>N*^*CA*^, **G-H)**. See also S1A and S1B Table. (**I-J**) Downregulation of Notch in TFC precursors (*bab1>N*^*RNAi*^, **I** and yellow in **J**) results in the appearance of TFs that contain fewer TFCs/TF. Upregulation of Delta in the soma (*bab1>Dl*, dark blue in **J**) causes the appearance of TFs with broadly distributed TFCs/TF numbers. The distribution of the TFs containing different TFC numbers is broader in mutants than in controls (**J**). See also S1B Table. (**A-C, E, G, I**) Images of the ovaries of control and mutant animals at the prepupal stage. TFCs are marked with En (green or white) and yellow brackets. CpCs are marked with En+Tj (yellow), ICs with Tj (red), germline is marked with Vasa (blue), and DAPI marks the nuclei (white). **(D, F, H, J)** The histograms visualize the frequency of observations for the TFC number per TF for the control and the mutants. The curves visualize the probability of the TFC number per TF based on the implied calculated normal distribution. In general, in *Notch* signaling mutants, the distribution of the TFs containing different numbers is broader than in controls. For statistical difference, a Kruskal-Wallis test was used. ***p≤0.001; **p≤0.01; *p≤0.05. See also S4F Fig and S1A and S1B Table.

TFs are similar in their organization to the stalk cells in adult ovaries. It has been previously shown that the length of stalks separating individual egg chambers corresponds to the amount of Notch signaling [50,51]. We tested if this is also the case for the TFs by altering Notch and Delta levels. Downregulation of the Notch receptor resulted in TFs containing fewer TFCs with abnormal shapes. Contrarily, overexpressing Delta caused more somatic cells to choose a TF cell fate: in general, individual TFs contained more TFCs (compare Figs 1D and 3B). Also, TFs of Notch mutants appeared to be abnormally shaped and exhibited cell intercalation defects (S4 Fig), which were similar to the Notch mutant phenotypes in stalks of adult ovaries [50,51]. Together, these data support the hypothesis that Notch signaling has a functional role in TF morphogenesis and that the number of cells accepting the TF cell fate is proportional to the extent of Notch signaling activation. However, it is not clear how the Notch signaling pathway is induced in TFC precursors, what is the source of Delta, and whether the germline communicates with the soma in the process of TF formation.

### Germline Delta activates Notch signaling in the posterior TFC precursors

Our examination of Notch signaling patterns in the developing TFs and analysis of Notch signaling mutants demonstrates that Notch signaling plays a role in the establishment of the TF cell fate and suggests that this fate can be induced via various Notch signaling modes that act in parallel: *(1)* germline Delta induces Notch signaling in the posterior TFCs and *(2)* somatic Delta represses Notch signaling in the anterior TFCs.

Firstly, to investigate the influence of germline Delta on TF formation, we overexpressed Delta specifically in PGCs. We closely examined individual TF organization in the prepupal ovaries and quantified the number of TFCs incorporated into each TF. Normally, there is not much variation in the number of TFCs per TF– 80% of TFs have exactly 8, and the other 20% have 7 or 9 TFCs incorporated into one TF stalk (Fig 3A–3J, green and blue curves, and S1A–S1C Table). This narrow distribution suggests that the process of TF formation is very robust. However, the increased expression of Delta in the germline had a dramatic effect on TF specification (Fig 3B). The number of TFCs per TF ranged from 4 to 14, and the average TFCs/TF number was significantly increased when compared to controls (Fig 3D, purple, and S1A Table). These data show that overexpression of Delta in the germline leads to an increase in TFC specification. This is consistent with previously published data demonstrating that the amount of Delta affects the strength of Notch signaling [23].

Secondly, to show that Delta from the germline plays a role in TF morphogenesis, we downregulated Delta specifically in the germline, which again resulted in a broader distribution in the number of TFCs per TF, where many TFs appeared to have reduced numbers of TFCs/TF (Fig 3C and 3D, magenta, and S1A Table). These data show that hypo-induction of Notch signaling caused by Delta downregulation in the germline decreases the number of somatic precursors adopting a TF cell fate.

Thirdly, to confirm that germline Delta is essential for TFC specification, we analyzed what happens in the absence of germline-produced Delta. In particular, we analyzed TFs in *tudor* maternal effect mutants that completely lack germline cells. Consistent with a previous study [33], we found that while TFs were formed in the germline-less ovaries, their appearance was dramatically impaired, and the numbers of TFCs per TF were significantly reduced (Fig 3E and 3F, orange, and S1A Table). These data show that germline Delta plays an instructive role, but it is not essential for TF assembly.

Fourthly, to test whether activation of Notch signaling in somatic cells is sufficient to induce TFC fate, we analyzed TFs in ovaries of transgenic animals expressing constitutively active Notch receptor under the control of *bab1-Gal4* (see the expression in S1F Fig). Consistent with the previous observation that the activation of Notch signaling coincides with the acquisition of TF cell fate, the expression of constitutively active Notch receptor in TFC precursors (*bab1-Gal4>UAS Notch*^*CA*^*)* led to the significant increase in TFCs per TF numbers (Fig 3G and 3H, pink, and S1B Table). These data show that ectopic activation of Notch signaling in ACs promotes TFC specification, suggesting that activated Notch is sufficient to induce TFC fate in a cell-autonomous manner.

Fifthly, since induction of Notch signaling via *trans-*activation requires the presence of the Notch receptor on the membranes of the signal-receiving cells, we quantified the number of TFCs/TF in *Notch-*deficient ovaries. In *Notch* (*N*^*ts1*^) temperature-sensitive mutants kept at the restrictive temperature (25°C) only during niche formation (L3), TFs appeared to be shorter with the numbers of TFCs per stalk distributed from 2 to 10 (Fig 3H, red, and S1B Table). This is significantly different from controls, *N*^*ts1*^ mutants kept at the permissive temperature (18°C) that, similarly to the wild type, had 7 to 9 TFC/TF (Fig 3H, dark green, and S1B Table). These data show that the presence of the Notch receptor in TFC precursors is important, but not essential, for TFC specification.

Together, our data on the germline-specific Delta manipulation and analyses of *tudor* and *Notch* mutants show that the TFC fate can be induced by two complementing mechanisms. Germline Delta induces TF cell specification via activation of Notch signaling in the posterior TFC precursors. This would require binding to and cleavage of the Notch receptor in the posterior TFC precursors. At the same time, high levels of non-activated somatic Delta inhibit Notch signaling in the anterior TFC precursors. Therefore, TFC precursor-specific deregulation of the Notch receptor or the Delta ligand in these cells should affect TFC specification. We tested this assumption by decreasing Notch receptor and increasing Delta ligand levels in stem cell niche precursors using a *bab1-Gal4* driver. We found that *Notch* downregulation (*bab1-Gal4>UAS Notch*^*RNAi*^) resulted in the appearance of TF stalks in which the numbers of incorporated TFCs was significantly decreased (Fig 3I and 3J, yellow, and S1B Table). These data show that even in the presence of germline Delta, TFC specification was affected when the levels of the Notch receptor were downregulated in TFC precursors (Fig 3I). In contrast, Delta ligand overexpression (*bab1-Gal4>UAS Delta*, Fig 3J, violet, and S1B Table) resulted in the appearance of TFs which contained significantly more TFCs in comparison to control (*bab1-Gal4>GFP*, Fig 3J, blue, and S1B Table) and in general a broader distribution of TFC/TF (from 2 to 14). These data show that even if the Notch receptor in soma and the Delta ligand in germline are normally expressed, ectopic Delta expression in the stem cell niche precursors affects their cell fate determination, resulting in abnormally formed TFs.

Together, these studies demonstrate that there are overlapping mechanisms to secure that the precursor cells acquire a certain Notch signaling status necessary for TF cell specification: inhibition of Notch signaling by Delta produced by somatic TFC precursors *per se* and activation of Notch signaling via distant induction by the germline-produced Delta ligand.

### The pattern of Notch signaling activation in TFC precursors is perturbed in Delta germline mutants

**Fig 4:**
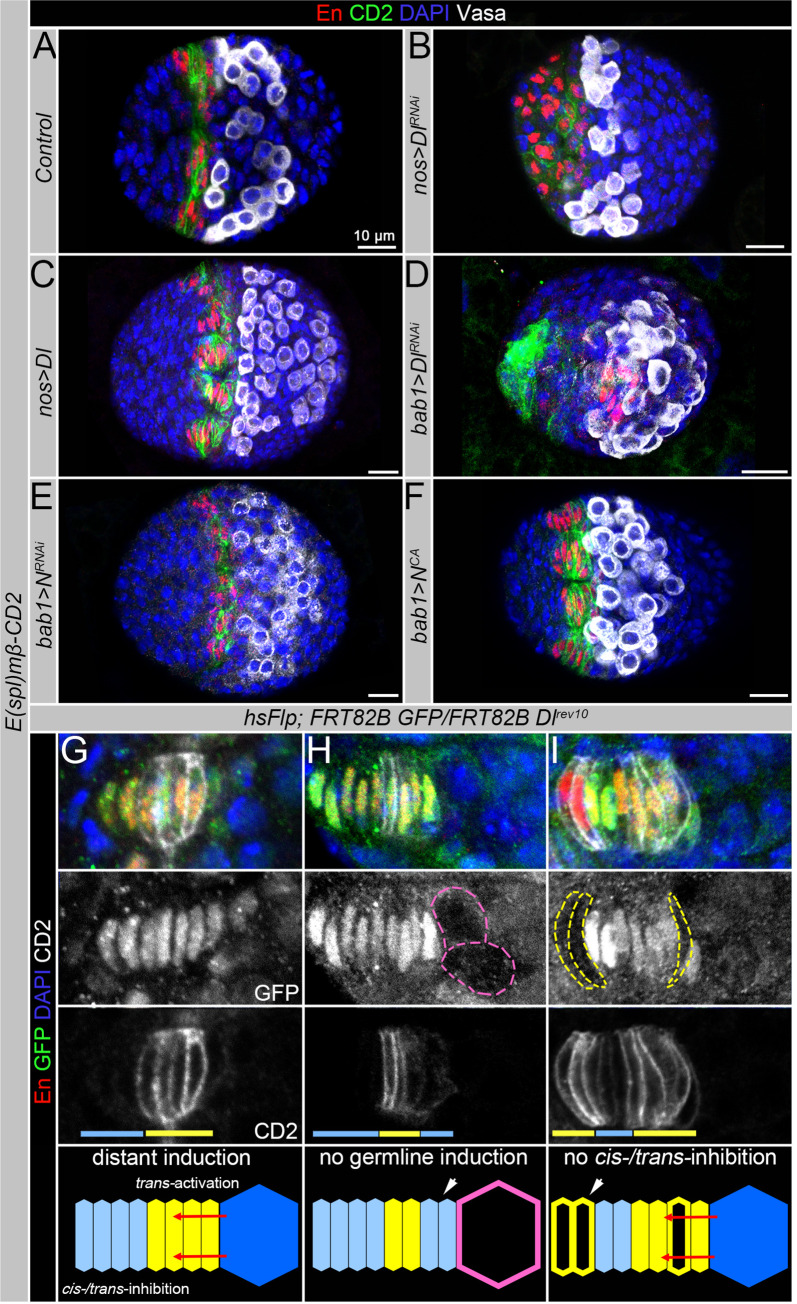
Perturbation of Notch signaling components in the germline or in somatic stem cell niche precursors alter the pattern of Notch signaling activation. **(A-D)** Expression of the Notch activity reporter (*E(Spl)mß-CD2*, green) and the stem cell niche marker En (red) in the developing ovaries of controls and various Notch signaling mutants. Vasa (white) marks the germline, DAPI (blue) marks nuclei. In the wild type (wt, **A**), the Notch activity reporter is activated in a disc-like fashion in the TFC precursors adjacent to the germline. However, when the germline-Delta is downregulated (*nos>Dl*^*RNAi*^, **B**), Notch reporter is induced in the more anterior TFC precursors in a “salt & pepper” pattern. Upon Delta upregulation in the germline, the Notch activity reporter is activated in TFC precursors more distal from the germline (*nos>Dl*, **C**). Downregulation of Delta expression in ACs allows ectopic activation of Notch signaling in the anterior somatic cells (*bab1>Dl*^*RNAi*^, **D**). Also, when the Notch receptor is downregulated in TFC precursors (*bab1>N*^*RNAi*^, **E**), the *E(spl)mβ-lacZ* reporter is irregularly activated in some of the TFC precursors. Expression of the constitutively active Notch receptor in TFC precursors (*bab1>N*^*CA*^, **F**) results in the forced activation of Notch signaling in all TFCs. (**G-I**) Analysis of the Notch activity reporter expression (*E(Spl)mß-CD2*, green) upon the clonal of induction of *Delta* loss-of-function mutations in the germline or soma. The cartoons are schematic models explaining Notch signaling activation modes. (**G**) In control TFs (no *Delta* clones), Notch signaling is activated in the TFCs adjacent to the germline (distant induction). (**H**) Upon loss of the germline Delta (GFP-negative cells outlined in pink), Notch signaling is activated in more anterior TFCs not adjacent to the germline (no germline activation). (**I**) When *Delta* clones are induced in the anterior TFCs (GFP-negative cells outlined in yellow), the Delta ligand no longer represses Notch activity, which results in the Notch signaling activation (no *cis-* or *trans-*inhibition). When *Delta* clones are induced in the posterior TFCs (black cells outlined in white), the Notch signaling activation pattern is not perturbed (*trans-*activation via germline Delta).

To further support the model that there are two mechanisms controlled by Notch signaling that play roles in the establishment of TF stalks, we analyzed the pattern of Notch activation in various mutants with perturbed expression of the Delta ligand and Notch receptor. According to the model, Notch signaling in the four posterior TFCs is activated by germline-derived Delta (Fig 4A); however, when Delta is downregulated in the germline, Notch signaling is not properly activated. Instead of inducing Notch activity in the somatic cells adjacent to the germline in a disc-like pattern, Notch signaling is randomly activated in the more anterior somatic cells in a “salt & pepper” pattern (Fig 4B, *nos>Dl*^*RNAi*^*)*. We propose that in the absence of germline Delta, somatic Delta plays a role in Notch signaling activation in the TFC precursors. Our expression analysis shows the Delta ligand as well as the Notch receptor are co-expressed in the TFC precursors, and it has been shown before that in a case when a population of cells co-expresses Notch and Delta, according to the rules of lateral inhibition, at least one cell has to shift the balance to express more Notch or Delta, which immediately will initiate the opposite (Delta or Notch) cell fate in the adjacent neighbor. Even in the Petri dish, cells will spontaneously self-organize into a reiterative “salt & pepper” pattern of Notch signal-receiving and Notch signal-sending cell fates [7,19,22,45]. Therefore, it is possible to activate Notch signaling and induce TF cell fate in some of the Delta-Notch co-expressing anterior somatic cells even without the instructions of the germline Delta.

Moreover, when Delta is upregulated in the germline, more Delta is produced and as a result, the Notch activity reporter is activated in a wider range of TFC precursors (Fig 4C, *nos>Dl*). However, when Delta is downregulated in ACs, Notch signaling is not only activated in the somatic TFC precursors adjacent to the germline, but in some ACs located more anteriorly that normally express high Delta needed to suppress Notch signaling (Fig 4D, *bab1>Dl*^*RNAi*^). It suggests that downregulating Delta expression in the anterior TFC precursors limits the ability of the highly expressed Delta ligand to inhibit Notch signaling.

Also, as expected, when the Notch receptor is downregulated in the Delta-receiving somatic TFC precursors, the *E(spl)mβ-CD2* reporter expression is weaker and has less distinct pattern when compared to control (Fig 4E, *bab1>N*^*RNAi*^). At the same time, the expression of the constitutively active Notch receptor in TFC precursors induced Notch signaling in all *bab1*-expressing TFCs (Fig 4F, *bab1>N*^*CA*^).

Together these data further support the hypothesis that the germline plays an instructive role in the induction of Notch signaling in the posterior TFC precursors via the *trans-*activation mode and that the somatic Delta inhibits Notch signaling in the anterior TFC precursors.

To strengthen this statement, we analyzed the pattern of Notch activation *(1)* in the TFs forming next to *Delta-*deficient PGCs and *(2)* in mosaic TFs containing wild type and *Delta-*deficient TFCs. When germline Delta was perturbed, the TFC precursors juxtapositioned to the mutant PGCs had dramatically reduced levels of *E(Spl)mß-CD2* expression, supporting the model that germline Delta plays an instructive role in turning Notch signaling “on” in the posterior TFC precursors (Fig 4G and 4H). When the Delta ligand was deleted in the anterior TFCs, it resulted in the activation of Notch signaling in these cells, which normally do not have Notch signaling “on” (Fig 4I), which supports the idea that the somatic Delta in the anterior TFC precursors inhibits Notch signaling via *cis-* or *trans-*inhibition. However, when the *Delta* mutation occurred in TFCs proximal to the germline, no obvious changes in Notch activity pattern were observed (Fig 4I), which is consistent with our hypothesis that the germline Delta induces Notch signaling in these cells. Importantly, regardless of the *Delta* clone position, the TF cell identity, as evaluated by the cylindrical cell shape and expression of the stem cell niche marker En, was not altered in *Delta*-deficient cells. These data additionally support the hypothesis that the TF cell fate can be induced by two different Notch signaling modes that act in parallel.

### The germline cells deliver Delta on projections to induce TF specification

Together, our sequential analysis of the Notch signaling activation pattern in the developing ovary and our genetic studies strongly suggest that it is the germline Delta expression that initiates stem cell niche formation. As mentioned before, Delta ligand expression was detected in the PGCs just prior to the beginning of TFC specification, and the first TF precursors that have Notch signaling activated and cell shape changed are the ones juxtapositioned to the germline, implying that the germline is the source of Delta that *trans-*activates Notch signaling in the ACs. For this type of Notch signaling activation, direct contact between the germline and TFC precursors is required. Since Notch signaling was activated several cell diameters away from the germline, we hypothesized that primordial germ cells could form projections that deliver Delta at a distance. We tested this hypothesis using several approaches.

**Fig 5:**
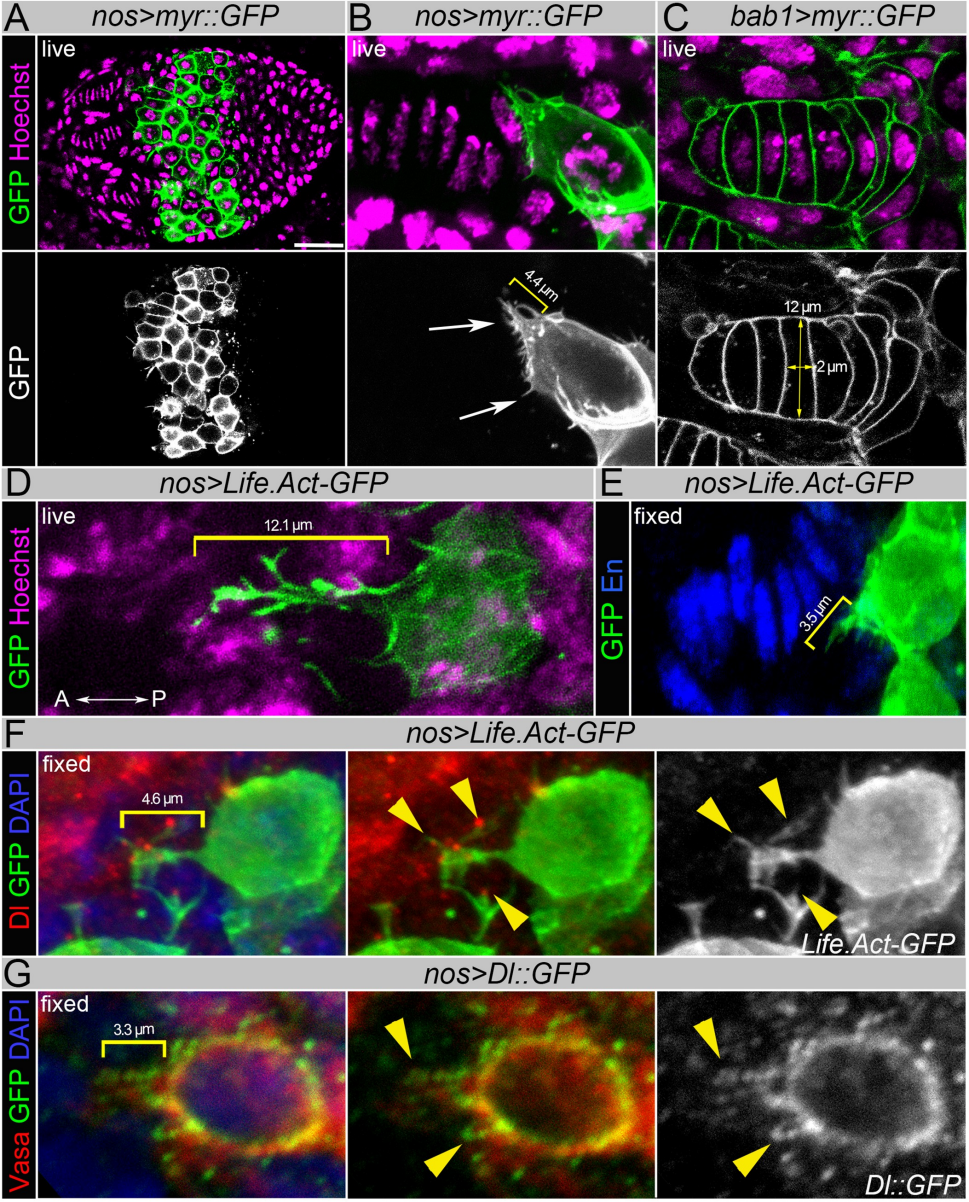
The germline cells deliver Delta on projections to induce stem cell niche formation. (**A-B**) Primordial Germline Cells (PGCs) form ultrafine projections (arrows) marked by membrane GFP (*nos>myr*::*GFP*) in live ovaries. (**C**) Note the absence of projections in the somatic cells of the germline stem cell niche (*bab1>myr*::*GFP*). Hoechst dye marks nuclei (magenta), GFP marks cell membranes (green). (**D-E**) Germline cell projections marked by *Life*.*Act-GFP* (*nos>Life*.*Act-GFP*) in both live (**D**) and fixed (**E**) ovaries. The brackets show the length of PGC projections directed towards TFs. Note that projections become shorter as a result of tissue fixation. GFP (green) marks the germline, Hoechst (magenta) marks nuclei in **D**, En (blue) marks TFCs in **D**. (**F**) Localization of Delta in germline projections in fixed ovaries (yellow arrowheads). Delta (red), GFP (green), DAPI (blue). (**G**) Expression analysis of the tagged version of Delta in the germline (*nos>UASp-Dl*::*GFP*) shows the presence of the Delta ligand on PGC extensions in fixed ovaries. Vasa (red), GFP (green), DAPI (blue).

Firstly, to test if cells in the developing ovary have any projections, we expressed membrane-bound GFP (*myr*::*GFP*) using the germline-specific *nos-Gal4* and somatic niche-specific *bab1-Gal4* drivers, which allowed the visualization of contours of the germline and somatic stem cell niche cells. Analysis in live ovaries showed that the PGCs can form ultrafine cellular projections (Fig 5A and 5B, arrows), while in the somatic niche cell these structures were not apparent (Fig 5C).

Secondly, to test if PGCs can form projections long enough to reach TFC precursors positioned 4–5 cells away from the germline, we checked the projection length in PGCs using *Life*.*Act-GFP* driven by the germline *nos-Gal4* driver. We found that these structures can be up to 12 μm long (Fig 5D), while the length of the TF is ~16–20 μm and the diameter and height of each cylindrical TFC is 9–12 μm and 1.5–3 μm, respectively (Fig 5C). These data demonstrate that the PGC can form cellular extensions which would allow them to communicate with somatic cells located even more than 4 cell diameters away. Supporting this hypothesis, it has been recently shown that adult GSCs can form various cellular projections to fine-tune Dpp signaling [52] and to reach and tightly ensheath CpCs [33].

Thirdly, to detect the presence of Delta on the germline projections, we performed immunohistostaining of the developing ovaries using anti-Delta antibodies (Fig 5G). As shown above, Delta is broadly expressed in the developing ovary (Fig 2A–2C). Upon tissue fixation, the ultrafine projections are largely destroyed (compare Fig 5D and 5E); however, we could distinctly detect the presence of the Delta ligand on the remaining parts of these microscopic structures (Fig 5F). To further test if Delta can decorate the germline cell projections, we expressed GFP-tagged Delta protein using a germline-specific driver (Fig 5G). Analysis of Delta subcellular distribution in the germline cells additionally confirms that the ligand Delta can be present on the germline cell projections. Together, these data demonstrate that PGCs can form cellular projections decorated with Delta that can extend for several cell diameters.

### Actin cytoskeleton plays a role in PGC projection formation

**Fig 6:**
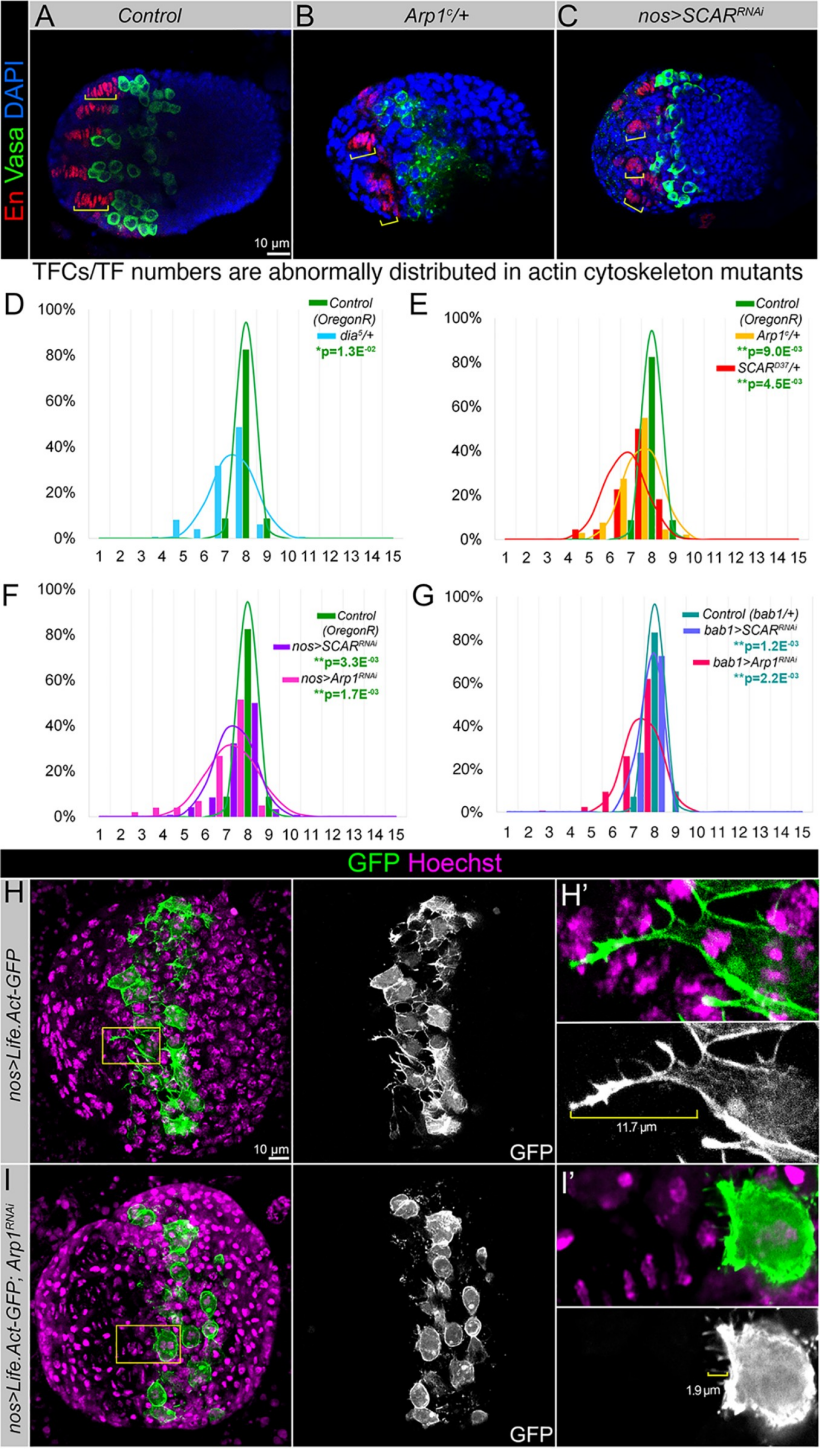
The length of TFs depends on actin dynamics. (**A-C**) Ovarian morphology is defective in actin cytoskeleton mutants. The brackets show the TF length in exemplary ovaries of the control (*Oregon R*, **A**), actin cytoskeleton heterozygous mutant (*Arp1*^*c*^*/+*, **B**), and mutants that have SCAR downregulated specifically in the germline (*nos>SCAR*^*RNAi*^, **C**). (**D-E**) Graphs represent the quantitative analysis of the variation of TFC numbers per TF. In actin cytoskeleton heterozygous mutants (*dia*^*5*^*/+*, *Arp1*^*c*^*/+*, *SCAR*^*d37*^*/+)*, the distribution of the TFs containing different numbers is significantly different than in control (green). (**F**) The number of TFC per TF is significantly decreased in mutants that have *SCAR* and *Arp1* downregulated specifically in the germline (*nos>SCAR*^*RNAi*^ and *nos>Arp1*^*RNAi*^). Note that downregulation of factors required for proper actin dynamics and cytoskeleton organization in specifically the germline significantly widens the distribution range of the TFC numbers. **(G)** The number of TFC per TF is significantly decreased in mutants that have *SCAR* and *Arp1* downregulated specifically in TFC precursors (*bab1>SCAR*^*RNAi*^ and *bab1>Arp1*^*RNAi*^). TFCs are marked by En (red), the germline is marked with Vasa (green), and nuclei are stained with DAPI (blue). The histograms depict the frequency of observations for the TFC number per TF for the controls and the mutants. The curves show the probability of the TFC number per TF based on the implied calculated normal distribution. For statistical difference, a Kruskal-Wallis test was used. **p≤0.01; *p≤0.05. See also S1C Table. (**H-I**) PGC cell projections marked by *Life*.*Act-GFP* in control (*nos>Life*.*Act-GFP*, **H**) and mutant ovaries that have the germline-specific downregulation of the actin-related protein (*nos>Life*.*Act-GFP; Arp1*^*RNAi*^, **I**) Note that in mutants, the germline projections are noticeably smaller. (**H’-I’**) show corresponding magnified images of yellow rectangles in the left panels. Hoechst dye marks nuclei (magenta), GFP marks cell membranes (green). The single-channel GFP images are shown in black and white. The brackets show the projection length.

To further demonstrate that the cellular projections generated by the germline play a role in ovarian stem cell niche assembly, we performed a pilot genetic screen. We studied mutants for cytoskeleton-maintaining factors, deregulation of which affects the formation of cellular projections. In particular, we focused on factors that have been shown to play a role in the *Drosophila* germline: *i)* Actin-related protein 1 (Arp1) which is the short filamentous component of the Dynactin complex that plays an essential role in the activation of the Dynein microtubule motor protein [53]; *ii)* a Wiskott-Aldrich Syndrome family protein (WASp) member, SCAR, which is an activator of the Arp2/3 complex that promotes actin polymerization and influences cell shape and motility [54]; and *iii)* a processive actin polymerase Diaphanous (Dia), which nucleates actin filaments *de novo*, stimulating actin addition at the barbed end and regulates cell shape change and projections [55]. Due to the lethality caused by the loss-of-function of these cell shape regulators, we analyzed their heterozygous mutants (Fig 6). We found that downregulation of any of these factors resulted in the significant alteration of the TFC number per TF. In general, TF had smaller numbers of TFCs, suggesting that actin dynamics affects TF assembly (Fig 6A–6E, blue, yellow, red, and S1C Table). Moreover, the germline-specific downregulation of the actin-binding protein SCAR and the actin-related protein Arp1 caused the appearance of short TFs, in which TFC numbers ranged from 3 to 9, in comparison to 8–9 in controls (Fig 6C–6F, violet, magenta, and S1C Table). This implies that the ability of the cytoskeleton to rearrange, specifically in the germline, plays an important role in TF formation.

It has also been shown that cellular projections generated by both communicating cells are important for proper signaling [56–59]. Therefore, we also perturbed actin cytoskeleton remodeling in the somatic stem cell niche cells using *Scar* and *Arp1* RNAi driven by *bab1-Gal4* (Fig 6G and S1C Table). Interestingly, the distribution of TFC numbers per TF was moderately but significantly altered when actin cytoskeleton remodeling was perturbed in the somatic TFC precursors. These data suggest that in the developing ovary, two-way signaling between the germline and soma exists and is communicated via cellular projections.

Finally, to see the significance of actin remodeling on germline projections, we studied their appearance in mutants with germline-specific downregulation of *Arp1* (Fig 6H–6I). Comparisons of PGC cell projections marked by *Life*.*Act-GFP* of control and actin germline mutants demonstrate that in mutants, the germline projections appear to be noticeably smaller (finer and shorter). These data imply that actin cytoskeleton remodeling is involved in the formation of cellular protrusions in the PGCs and that these protrusions play a role in germline-to-soma communications, establishing the specific signaling pattern necessary for proper cell niche assembly. Combined, these results demonstrate that to induce Notch signaling, the Delta ligand produced by the germline is delivered via actin-mediated cellular projections to the somatic precursor cells positioned several cell diameters away from the Delta source. This is an example of Notch signaling activation among non-equipotent cells via a distant induction mode.

## Discussion

**Fig 7:**
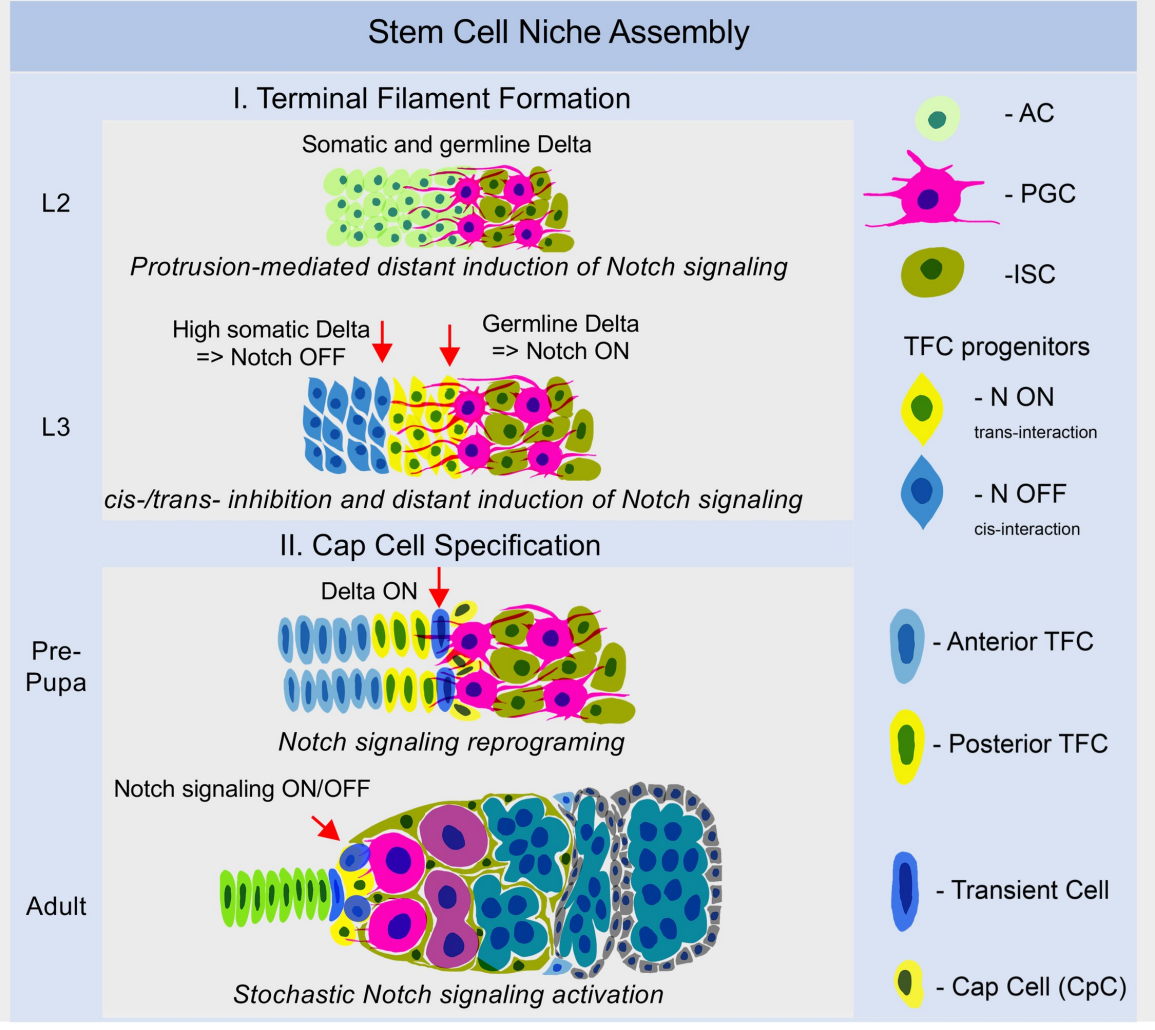
Schematic depicting differentiation events occurring during germline stem cell niche formation. In the process of stem cell niche assembly, two distinct steps can be described–TF filament formation and CpC specification–both of which depend on Notch signaling activation. During early stages, anterior cells (AC, light green) express both Delta and Notch and are undecided. At EL3, the Delta ligand expressed on the projections of primordial germ cells (PGCs, magenta) *trans-*interacts with the Notch receptor on the membrane of posterior TFC precursors (yellow cells), resulting in the Notch signaling activation in these cells. They acquire TFC fate, which leads to alteration of their shape, cell intercalation, mechanical tissue rearrangement, and formation of separate stalks. Anterior TFC progenitors (blue cells) are too distant to have Notch signaling activated by germline Delta. However, they express Delta that can bind Notch intrinsically or extrinsically at their own or at juxtaposed cell membranes, resulting in Notch signaling inhibition in *cis* or *trans*. Thus, a double mechanism of Notch signaling acquisition ensures TF formation. Later, the posterior TFC called Transition Cell (dark blue) is reprogrammed into Delta-sending to induce the CpC fate in six adjacent stem cell niche precursors. Finally, during adulthood, cells of the stem cell niche exhibit stochastic fluctuations in Notch signaling activity to sustain the niche functions, which guarantees that the germline stem cells will have their lifetime residence and will continue to divide, thereby securing progressive oogenesis. Thus, the acquisition of a certain status of Notch signaling is key for niche cells’ terminal differentiation and function as a lifetime support for germline stem cells.

Here we show that the formation of the germline stem cell niche in *Drosophila* depends on the accuracy of spatial patterning within the developing organ, which is controlled by differential activity of Notch signaling (Fig 7). In particular, extrinsic induction (*trans-*activation) of Notch signaling in TFC precursors by the germline-produced Delta ligand regulates the key step of ovarian morphogenesis, TFC fate acquisition, which leads to the assembly of individual TF stalks. The germline acts as a source of Delta that activates Notch in the adjacent anterior somatic cells; moreover, cellular projections allow refinement of the Notch signaling pattern. They are formed by primordial germ cells and sent several cell diameters to specify TFCs. Additionally, the Notch-Delta interaction between anterior TFC precursors also contribute to TFC specification. Thus, to initiate the germline stem cell niche formation, Notch signaling is patterned by two different mechanisms that act in parallel. Later, TFs also act as Notch-signal sending centers to activate Notch signaling in GSC niche cell precursors, which requires that the status of Notch signaling in one of the posterior TF cells (Transition Cell, dark blue, Fig 7) is reprogrammed from the Notch signal-receiving to Delta-expressing cell [31]. Thus, our data show that during the process of *Drosophila* ovarian morphogenesis, multiple layers of Notch signaling pathway activation define the specificity of various niche cell fates in the process of the stem cell niche unit assembly.

Apparently, the acquisition of a certain status of Notch signaling (ON/OFF, low/high) is key for niche cells’ terminal differentiation and function as a lifetime support for germline stem cells. Notch signaling is predominantly considered to act as short-range juxtacrine signaling between adjacent cells [11,60]. Here we also see that Notch can be categorized as medium-range signaling used for cell-to-cell communications, where the Delta ligand plays the role of a distant signaling factor. It is produced by a Notch signal-sending cell, and its interaction with the Notch receptor in the nearby and distant cells results in a change in their cell fate. There are several critical steps in posttranslational processing of the Delta ligand necessary for its full activation [61]; however, it is not clear what would affect the activity of Delta as a distant signaling ligand. Recent findings increasingly suggest that cells directly communicate with each other via cellular projections to ensure effectiveness and selectivity of cell-to-cell signaling [62–65]. In particular, they have been shown to be important for BMP signaling communication between *Drosophila* GSC and their niches [52,66]. Dynamic filopodia in the developing wing disk that are induced by Delta expression allow refinement of the distant Notch signaling pattern [12,13]. Here we demonstrate that the germline-produced Delta can induce a Notch active cell state in the somatic cells that are at least 4 cell diameters away from the Delta source and that the germline cells form extended projections that are decorated with the Delta protein. The Delta ligand is delivered to the membranes of the posterior TFC precursors via these subcellular structures to initiate stem cell niche formation. It would be important to study this process further and identify the type and dynamics of these Delta-positive projections.

Usually, Notch signaling is required to induce different cell fates among equipotent precursor cells. It has also been demonstrated that cells of different origin can communicate via Notch signaling. For example, germline-expressed Delta in the developing oocyte can activate Notch signaling in the adjacent follicular epithelial cells, which induces the mitotic-to-endocycle switch in these cells adjacent to the Delta source [42]. However, it has not been shown before that the distant induction of Notch signaling can occur among non-equipotent cells, where one cell type sends Delta ligand across several cell diameters to cells of another origin, such as the germline and somatic cells in the developing gonad.

Intriguingly, we also found that the TFC fate can be induced via two different Notch signaling modes. The GSC niche unit must be positioned next to the germline to induce GSC fate in a group of primordial germ cells; therefore, it is logical that the germline itself is instructive in this process. However, even in germline-less ovaries, TFs and the niche are established, which supports the idea that there is a second mechanism for the induction of TF cell fate. In particular, somatic anterior cells co-express Delta and Notch, which interact in *cis* or in *trans*, leading to Notch inhibition in these cells. Apparently, both Notch activation and Notch inhibition in anterior somatic cells allows them to become TF cells. Similarly, we have recently described that a second mechanism that secures Notch signaling activation also exists to ensure the robustness of stem cell niche assembly [31]. Normally, steroid signaling induces TFC reprogramming into a Delta-sending cell that can induce the perfect hexagonal Notch-signaling pattern in the adjacent CpC precursors via the local induction mechanism (Fig 1A). In addition, Notch signaling can be activated via lateral inhibition, which occurs among the equivalent cell populations. In this case, stem cell niche precursor cells (ICs) are bivalent and co-express both the Delta ligand and Notch receptor. They can acquire Notch active status stochastically, since even without inductive signaling, fluctuations in Delta expression permit one random cell to win and induce Notch signaling in the adjacent cells, which converts them into niche cells and results in the appearance of ectopic niches [31]. Therefore, the niche precursor cell bistability can be resolved by two mechanisms of Notch signaling activation: peripheral local induction and lateral inhibition, which is extrapolated onto the patterning of the adult stem cell niche.

Interestingly, even though posterior TFCs and CpCs both exhibit active Notch signaling, the intensity of Notch activation is different in these cells: TFCs have high, while CpCs have low Notch activity. Recently it has been proposed that the geometry of cell-to-cell contacts influences long- and short-range signaling and diversifies signaling patterns [21]. This work assumes that the kinetics of Notch-Delta signaling depend on junctional contacts, which are different at the projections and at the cell interfaces. In the case of the current study, these would be the contacts between the Delta-positive germline-generated projections and TFC precursors, or between the Delta-sending reprogrammed TFC and CpC precursors. Moreover, our analyses of actin mutants suggest that that the two-way signaling communicated via cellular projections exists between the germline and somatic cells in the developing ovary. It has been shown previously that cellular projections control various processes, from morphogenesis to tumorigenesis, via the distribution of signaling proteins [56,59,67–69]; thus, it is important to study further the activity of cellular projections to understand how Notch signaling activation depends on the projection-mediated dynamics between the germline and the soma.

Together, these data show that multiple modes of Notch signaling activation indicate the complexity of spatial cellular Delta-Notch interactions, resulting in differential Notch signaling activation, tissue patterning, and maintenance. Even though it takes some developmental time, important is that all TFCs and CpCs eventually acquire a certain Notch signaling status. This promotes establishment of a stable pattern and commitment to a fixed fate, which eventually allows the stem cell niche formation. At the same time, during adulthood, GSC niche cells have fluctuating and possibly self-organizing Notch ON/OFF signaling patterns, which is important, since niche cells are essential for a life-long maintenance of GSCs and must be able to sustain their own cell fate and signaling status. Potentially, bioengineering of self-maintaining stem cell niches for *in vivo* engraftment, maturation and maintenance of stem cells, and also differentiation niches for governance of the efficiency of stem cell progeny differentiation would be of great advantage for regenerative medicine. In addition, a better understanding of stem cell niches in their natural environment may aid the understanding of the development of many diseases.

## Materials and methods

### Fly strains and genetics

Fly stocks were maintained at 25°C on a standard cornmeal-agar diet in a controlled environment (constant humidity and light-dark cycle) unless otherwise stated. As Control, *OregonR* crossed to *w*^*1118*^ line was used.

To manipulate Notch signaling, *UASp-Dl* [23], *UAS-Notch*^*CA*^ [23,51], *UASt-Dl-RNAi* [Bloomington Drosophila Stock Center (BDSC) 36784], *UASt-Dl* (BDSC 26695), *UASt-N*^*RNAi*^ (BDSC 27988) and *N*^*ts1*^ (gift from Frank Hirth) lines were used. To downregulate Notch levels, *N*^*ts1*^ mutants were kept at a semi-restrictive temperature (25°C) through all stages of development. For ectopic expression in the germline or soma, the following driver lines were used: *nanos-Gal4* (*nos-NGT-Gal4*; *VP16-nos*.*UTR-Gal4*; BDSC 4442, BDSC 4937) or *bab1-Gal4/TM6* [70], BDSC 6803). To mark germline projections, *UASp-Life*.*Act-mGFP* (BDSC 58718) and *UAS myr*::*GFP* (BDSC 77124) lines were used. To induce Delta loss-of function clones, *FRT 82B Dl*^*rev10*^*/TM3*, *Sb* and *hs Flp; FRT 82B Ubi-GFP* (BDSC 5188) lines were used [71]. To visualize Notch signaling activity, Notch activity reporters *E(Spl)mß-CD2* [gift from Wu-Min Deng [43]] and *NRE-GFP* (BDSC 30727) were used. To visualize Delta, *UAS-Dl*::*GFP* lines were used (BDSC 8610 and 8611). To disturb actin cytoskeleton organization, the following mutants were used: *Arp1*^*c*^ (BDSC 11424), *SCAR*^*d37*^ (BDSC 8754), *dia*^*5*^ (BDSC 9138), *Arp1*^*RNAi*^ (BDSC 67932) and *SCAR*^*RNAi*^ (BDSC 51803) To obtain ovaries without germline, female progeny from homozygous *tudor* mutant (*tud*^*B42*^, gift from Ruth Lehmann) were analyzed.

### Staging of ovaries

For EL3 stage (72 h AEL), larvae that match criteria of L3 size but still feeding were picked. For LL3 stage (~112 h AEL), larvae that started wandering and were out of the food were picked. For prepupal stages (120h AEL), puparia that were still white but had distinct puparium shape and not moving anymore were picked.

### Immunohistochemistry

Ovaries were dissected in phosphate-buffered saline (PBS) and fixed while shaking on a nutator for 15 min in PBS containing 4% formaldehyde. Next, they were rinsed with PBT (PBS/0.2%, Triton X-100) four times (15 min, each rinse) and blocked in PBTB (PBT, 0.2% BSA, 5% Normal Goat Serum) for 1 h at room temperature. The tissue was incubated with primary antibodies overnight at 4°C. The next day, they were rinsed with PBT four times (15 min, each rinse) and blocked in PBTB for 1 h at room temperature. The ovaries were then incubated in secondary antibodies overnight at 4°C. The next day, they were rinsed with PBT (4 times, 15 min each rinse) and stained with DAPI (1 mg/ml in PBT) for 10 min. Finally, they were washed with PBT twice (5 min, each wash) and dissected onto slides in 70% glycerol, 3% NPG, 1× PBS [72]. For live analysis of germline projections, ovaries were dissected in PBS, stained with Hoechst and immediately imaged under the confocal microscope.

The following primary antibodies were used: mouse anti-Engrailed (En; 1:20), mouse anti-N^ICD^ (1:20) and mouse anti-Delta (Dl, 1:20) from Developmental Studies Hybridoma Bank, rabbit anti-Vasa (1:5000, gift from Herbert Jäckle), mouse anti-CD2 (1:100, Biolegend), guinea pig anti-Traffic Jam (Tj, 1:5000, gift from D. Godt), chicken anti-GFP (1:5000, Abcam). Secondary antibodies: goat anti-rabbit Alexa 488, goat anti-chicken Alexa 488, and goat anti-guinea pig Alexa 647 (1:500, Life Technologies), goat anti-mouse Cy3 IgG1 and goat anti-mouse Alexa 488 (1:250, Jackson ImmunoResearch Laboratory). For visualizing cell nuclei, DAPI (Sigma) and Hoechst (Thermo Fisher) dyes were used. Samples were analyzed using a confocal microscope (Zeiss LSM 700). For making figures, Adobe Photoshop, Adobe Illustrator and Sketches software were used.

### Visualization of cellular projections in live ovaries

Transgenic animals expressing membrane GFP—*UASp-Life*.*Act-mGFP* (BDSC 58718) or *UAS myr*::*GFP* (BDSC 77124) under control of *nanos-Gal4* or *bab1-Gal4* drivers were generated. To visualize the GFP expression in the germline or in the somatic cells of the stem niche, prepupal ovaries were dissected in Schneider’s medium. To visualize nuclei, 2 μg/ml solution of Hoechst 33258 (bisbenzimide) DNA stain in Schneider’s medium was used. After 5 min of Hoechst staining, live dissected ovaries were placed on a slide with a drop of Halocarbon oil 27 (Sigma Aldrich), covered with a coverslip, and immediately subjected to confocal imaging.

### Analysis of the Notch signaling activity in TFs

Prepupal ovaries of transgenic animals containing Notch activity reporters–*E(Spl)mß-CD2* or *NRE-GFP*–were dissected, fixed, and stained with anti-Delta and anti-GFP antibodies, and DAPI. Fluorescent intensity peaks of the Delta receptor and the Notch signaling activity reporter (GFP) in TFCs were produced using ZEN Lite software.

### Analyses of the TFC numbers

To analyze the number of TFCs/TF, Z-stack confocal images of the entire ovary at prepupal stage (120h AEL) with 1 μm intervals were captured. TFCs were identified by the disk-shaped morphology and En expression. Firstly, we quantified the number of TFC per TF in controls and different mutants, which varied from 1 to 15. Secondly, the mean and standard deviation of the number of TFC per TF per ovary of control and the mutant was calculated. Thirdly, a Kruskal-Wallis test was performed to test for difference in means between the control and the mutant. Finally, in order to visualize the difference, each mutant was plotted against the control by calculating the probability of the TFC number per TF using the mean and standard deviation derived from the quantified numbers of TFC per TF in controls and mutants.

## Supporting information

S1 Fig In the developing ovary, the expression patterns of the Notch receptor and the Delta ligand are dynamic. (**A**) At EL3 stage, the Notch protein is present in all somatic cells. (**B**-**C**) As development progresses, at LL3 and Prepupa stages, Notch expression becomes more pronounced in anterior cells (AC, red arrows) and less noticeable in TFC precursors and TFCs, (cyan arrows). Since TF precursor cells express both Notch and Delta at earlier stages, the absence of Notch staining plausibly occurs due to the interaction between Delta and Notch, leading to Notch signaling activation (after the Notch receptor is cleaved at the membrane, the amount of intracellular Notch translocated to the nucleus is too small to be detected by anti-N^intra^ antibodies). Notch receptor expression is also undetectable CpCs (yellow arrows) at Prepupa stage. Notch (magenta), Vasa (green), DAPI (blue). (**D-E**) To verify the specificity of the anti-Dl antibody staining in the germline, Dl expression was downregulated using *UAS-Dl*^*RNAi*^ driven by *nanos-Gal4* (*nos>Dl*^*RNAi*^*)*. In comparison to OregonR (*wt*), Dl protein expression levels are notably downregulated in the PGCs of *nos>Dl*^*RNAi*^ mutants (magenta arrows). Note, Dl expression is not changed in the anterior somatic cells of these mutants in comparison to controls (red arrows). Delta (red), Vasa (green), DAPI (blue). (**F**) Pre-adult ovarian expression patterns of the somatic *bab1-Gal4* driver visualized by the membrane GFP (*bab1>CD8*::*GFP*). Cyan arrows indicate TFCs, yellow arrows indicate CpCs. En (red), GFP (green), DAPI (blue), Vasa (white).
(TIF)

S2 Fig Notch signaling activation is differentiation stage-dependent. (**A**) Expression of the Notch activity reporter (*E(spl)mβ-CD2*, green) and Delta protein (red) in the TF at the prepupal stage (P1). Peaks on the graph below represent the fluorescence intensity of the Notch activity reporter and Delta protein in the TFCs from the upper panel. Graph shows activation of Notch signaling in posterior TFCs in response to the germline Delta (*trans*-activation). Lower panels show Delta protein and Notch activity reporter expression in single channels. Anterior TFCs have Delta and no Notch activity, which is consistent with Notch signaling inhibition mode by *cis-* or *trans-*Delta. Note the absence of Notch activity in the most posterior TFC (Transition Cell), which is reprogrammed into a Delta-sending cell via steroid-induced *miR-125* [31]. (**B**) Expression of another Notch activity reporter (*NRE-GFP)* in the TF at the prepupal stage. Peaks on the graph represent the fluorescence intensity of the reporter. Anterior ↔ Posterior (A↔P).
(TIF)

S3 Fig Expression of the Notch signaling reporter in adult germarium. (**A**) Schematic drawing of adult germarium: Germline Stem Cell (GSC, magenta), Cap Cell (CpC, yellow and blue), Transition Cell (TC, blue), Terminal Filament Cell (TFC, green), Escort Cell (olive), Cystoblast (plum), Cyst (teal), Follicular epithelium (grey), Follicular Epithelium Stem Cell (light blue). (**B**) Expression of the Notch activity reporter (*E(spl)mβ-CD2*, red) in adult germarium. Note that Notch reporter is present in some CpCs (arrows) and TFCs (arrowheads). CD2 (red), Vasa (green), DAPI (blue).
(TIF)

S4 Fig Notch is involved in the first step of GSC niche assembly, the process of TF formation. (**A-E**) Notch signaling manages proper TF assembly. Examples of TFs with abnormal lengths and cell numbers (brackets) observed upon Notch downregulation (*N*^*1ts*^, 25°C during L3, **B**), germline Delta upregulation (*nos>Dl*, **C**), and somatic Notch downregulation (*bab1>N*^*RNAi*^, **D**). TFCs are marked with En (green), CpCs are marked with En+Tj (yellow), ECs are marked with Tj (red), the germline is marked with Vasa (blue). En and Tj are also shown in a separate channel in white. (**F**) Notch signaling manages proper number of TFCs per TF. Box plots represent the quantitative analysis of the TFC numbers per TF. Note that deregulation of Notch signaling significantly affects the distribution range of the TFC numbers. F-test was used to test for statistical significance: ***P≤0.001. (**G**) Notch signaling promotes TFC intercalation. Bar graphs represent the percentage of the observed atypical TF phenotypes caused by abnormal TFC intercalation (n>100 TFs, at least three biological replicates). Note that “not-intercalated” TFs in Notch signaling mutants also contain abnormal TFCs/TF numbers, suggesting that Notch signaling controls TFC specification and TF assembly in parallel. Two-way tables and χ^2^-test were used to test for statistical significance ***P≤0.001.
(TIF)

S1 Table A: The germline influences the number of Terminal Filament Cells per individual Terminal Filament (TFCs/TF); B: Notch signaling controls the number of Terminal Filament Cells per individual Terminal Filament (TFCs/TF); C: Actin dynamics control the number of Terminal Filament Cells per individual Terminal Filament (TFCs/TF).
(DOCX)
