# Supplementary figures and images for: Distant activation of Notch signaling induces stem cell niche assembly
Source: PLoS Genet. 2021 Mar 29;17(3):e1009489. doi: 10.1371/journal.pgen.1009489 (PMC8031783; doi:10.1371/journal.pgen.1009489)

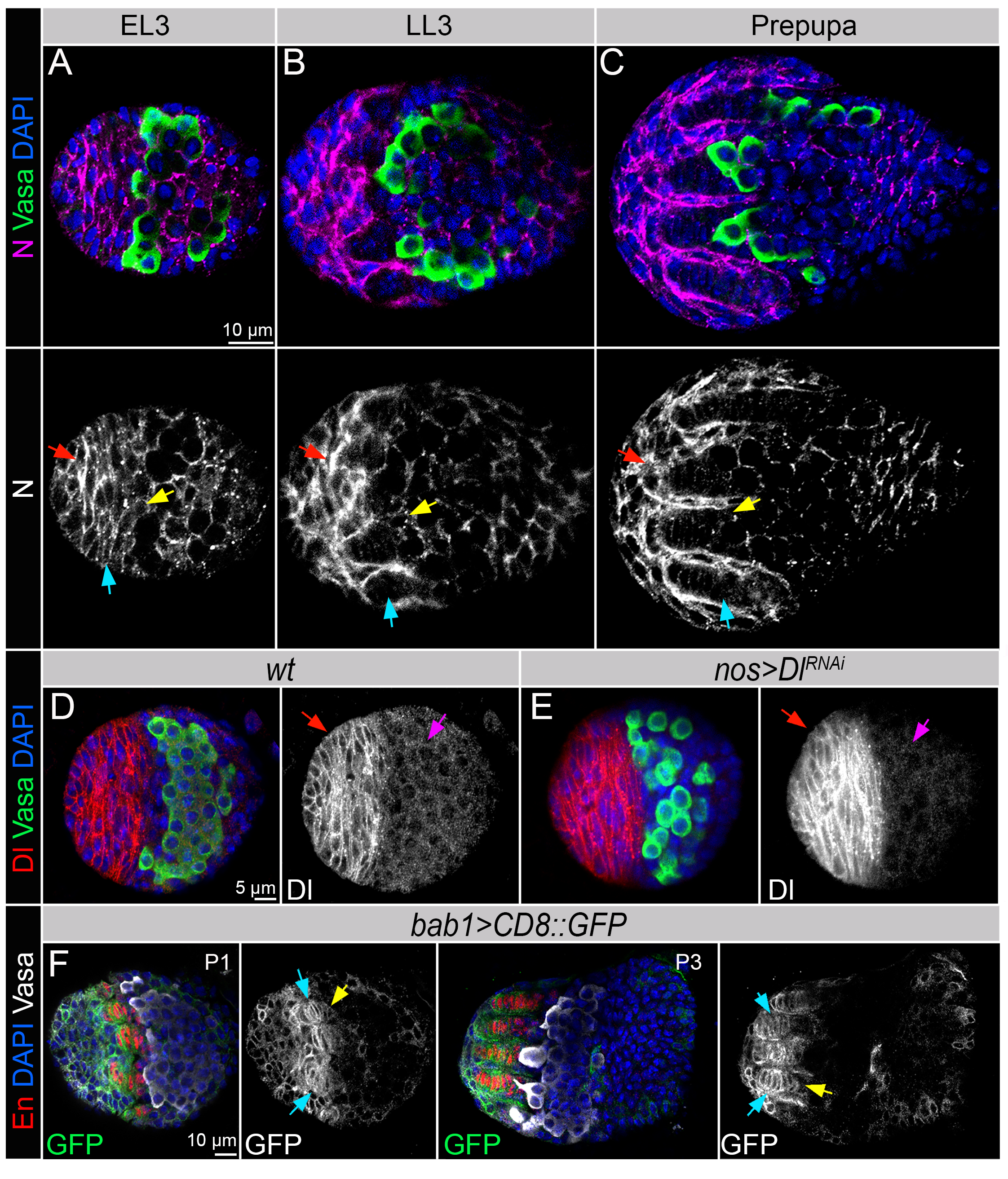

Supplement: S1 Fig — (A) At EL3 stage, the Notch protein is present in all somatic cells. (B-C) As development progresses, at LL3 and Prepupa stages, Notch expression becomes more pronounced in anterior cells (AC, red arrows) and less noticeable in TFC precursors and TFCs, (cyan arrows). Since TF precursor cells express both Notch and Delta at earlier stages, the absence of Notch staining plausibly occurs due to the interaction between Delta and Notch, leading to Notch signaling activation (after the Notch receptor is cleaved at the membrane, the amount of intracellular Notch translocated to the nucleus is too small to be detected by anti-Nintra antibodies). Notch receptor expression is also undetectable CpCs (yellow arrows) at Prepupa stage. Notch (magenta), Vasa (green), DAPI (blue). (D-E) To verify the specificity of the anti-Dl antibody staining in the germline, Dl expression was downregulated using UAS-DlRNAi driven by nanos-Gal4 (nos>DlRNAi). In comparison to OregonR (wt), Dl protein expression levels are notably downregulated in the PGCs of nos>DlRNAi mutants (magenta arrows). Note, Dl expression is not changed in the anterior somatic cells of these mutants in comparison to controls (red arrows). Delta (red), Vasa (green), DAPI (blue). (F) Pre-adult ovarian expression patterns of the somatic bab1-Gal4 driver visualized by the membrane GFP (bab1>CD8::GFP). Cyan arrows indicate TFCs, yellow arrows indicate CpCs. En (red), GFP (green), DAPI (blue), Vasa (white). (TIF) [file pgen.1009489.s001.tif]

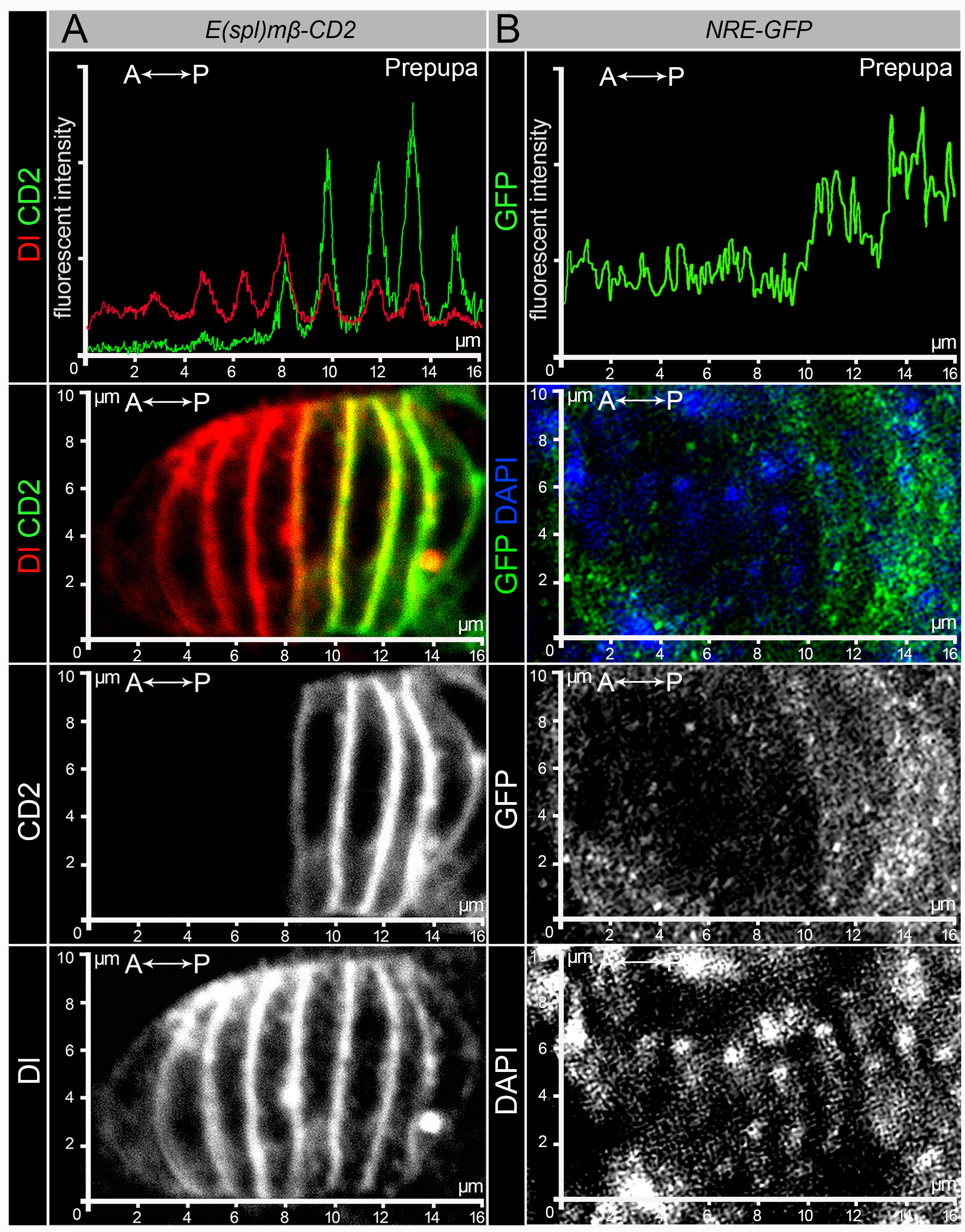

Supplement: S2 Fig — (A) Expression of the Notch activity reporter (E(spl)mβ-CD2, green) and Delta protein (red) in the TF at the prepupal stage (P1). Peaks on the graph below represent the fluorescence intensity of the Notch activity reporter and Delta protein in the TFCs from the upper panel. Graph shows activation of Notch signaling in posterior TFCs in response to the germline Delta (trans-activation). Lower panels show Delta protein and Notch activity reporter expression in single channels. Anterior TFCs have Delta and no Notch activity, which is consistent with Notch signaling inhibition mode by cis- or trans-Delta. Note the absence of Notch activity in the most posterior TFC (Transition Cell), which is reprogrammed into a Delta-sending cell via steroid-induced miR-125 [31]. (B) Expression of another Notch activity reporter (NRE-GFP) in the TF at the prepupal stage. Peaks on the graph represent the fluorescence intensity of the reporter. Anterior ↔ Posterior (A↔P). (TIF) [file pgen.1009489.s002.tif]

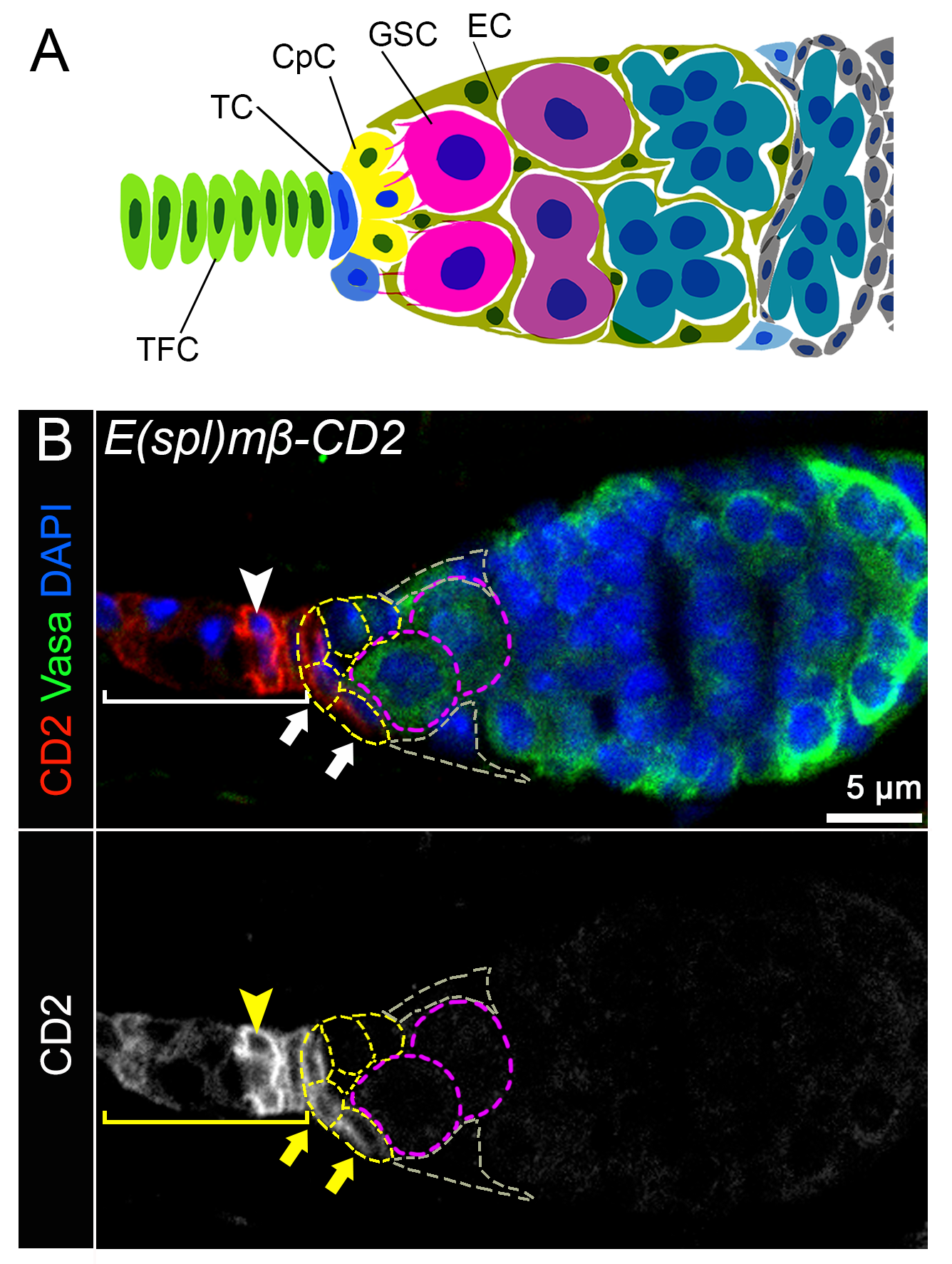

Supplement: S3 Fig — (A) Schematic drawing of adult germarium: Germline Stem Cell (GSC, magenta), Cap Cell (CpC, yellow and blue), Transition Cell (TC, blue), Terminal Filament Cell (TFC, green), Escort Cell (olive), Cystoblast (plum), Cyst (teal), Follicular epithelium (grey), Follicular Epithelium Stem Cell (light blue). (B) Expression of the Notch activity reporter (E(spl)mβ-CD2, red) in adult germarium. Note that Notch reporter is present in some CpCs (arrows) and TFCs (arrowheads). CD2 (red), Vasa (green), DAPI (blue). (TIF) [file pgen.1009489.s003.tif]

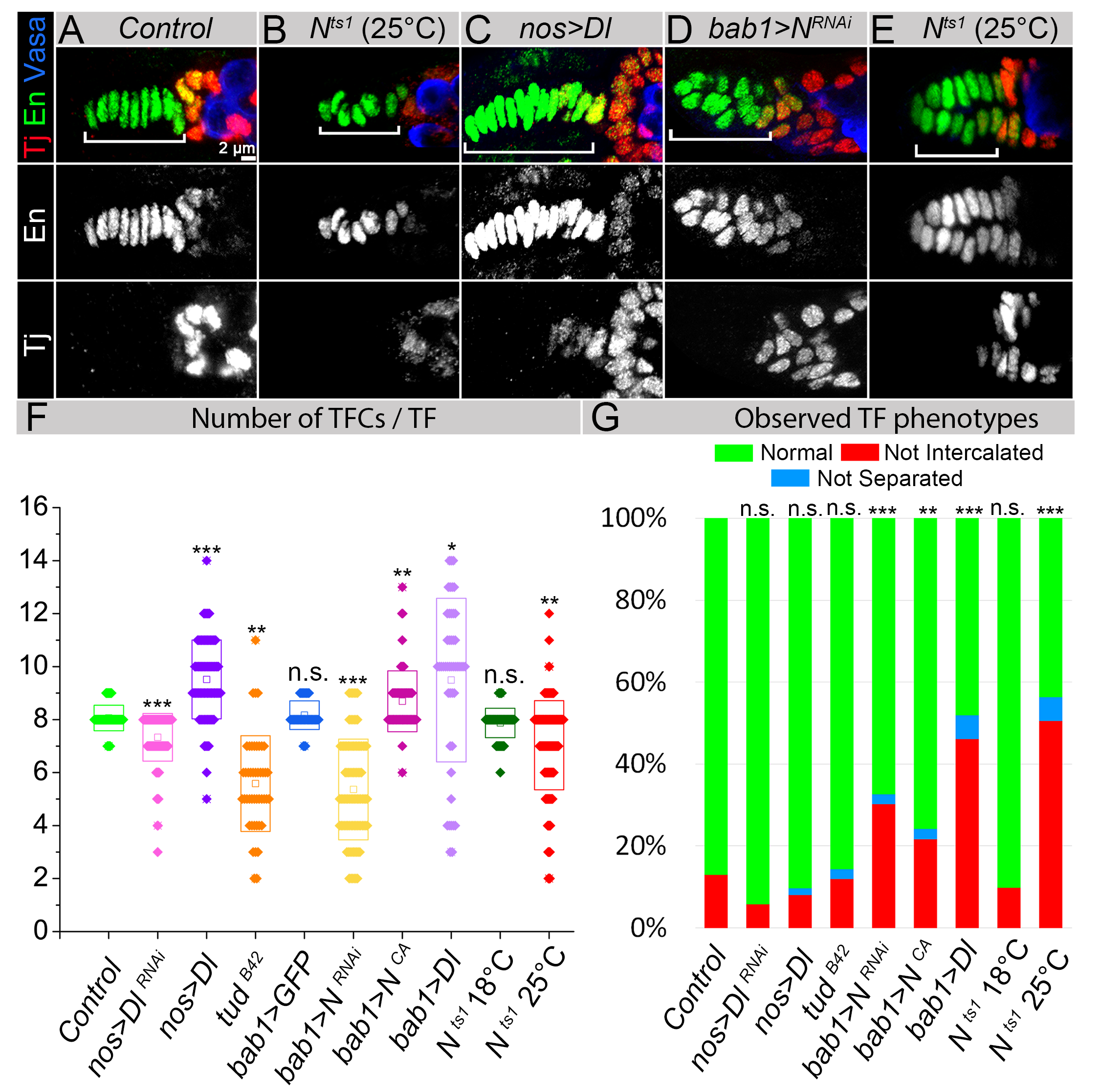

Supplement: S4 Fig — (A-E) Notch signaling manages proper TF assembly. Examples of TFs with abnormal lengths and cell numbers (brackets) observed upon Notch downregulation (N1ts, 25°C during L3, B), germline Delta upregulation (nos>Dl, C), and somatic Notch downregulation (bab1>NRNAi, D). TFCs are marked with En (green), CpCs are marked with En+Tj (yellow), ECs are marked with Tj (red), the germline is marked with Vasa (blue). En and Tj are also shown in a separate channel in white. (F) Notch signaling manages proper number of TFCs per TF. Box plots represent the quantitative analysis of the TFC numbers per TF. Note that deregulation of Notch signaling significantly affects the distribution range of the TFC numbers. F-test was used to test for statistical significance: ***P≤0.001. (G) Notch signaling promotes TFC intercalation. Bar graphs represent the percentage of the observed atypical TF phenotypes caused by abnormal TFC intercalation (n>100 TFs, at least three biological replicates). Note that “not-intercalated” TFs in Notch signaling mutants also contain abnormal TFCs/TF numbers, suggesting that Notch signaling controls TFC specification and TF assembly in parallel. Two-way tables and χ2-test were used to test for statistical significance ***P≤0.001. (TIF) [file pgen.1009489.s004.tif]
