# Supplementary material for: Distant activation of Notch signaling induces stem cell niche assembly
Source: PLoS Genet. 2021 Mar 29;17(3):e1009489. doi: 10.1371/journal.pgen.1009489 (PMC8031783; doi:10.1371/journal.pgen.1009489)
Supplement: S1 Table — A: The germline influences the number of Terminal Filament Cells per individual Terminal Filament (TFCs/TF); B: Notch signaling controls the number of Terminal Filament Cells per individual Terminal Filament (TFCs/TF); C: Actin dynamics control the number of Terminal Filament Cells per individual Terminal Filament (TFCs/TF). (DOCX) [file pgen.1009489.s005.docx]

**S1A Table. The germline influences the number of Terminal Filament Cells per individual Terminal Filament (TFCs/TF)**

| **Possible TFCs/TF**  **numbers** | **Probability of various TFCs/TF numbers**  **based on the observations in different mutants** | | | |
| --- | --- | --- | --- | --- |
| 1 | 9.5E-48 | 2.0E-08 | 7.0E-12 | 8.8E-03 |
| 2 | 2.2E-35 | 7.5E-07 | 9.8E-09 | 3.1E-02 |
| 3 | 6.9E-25 | 1.8E-05 | 3.9E-06 | 7.9E-02 |
| 4 | 2.7E-16 | 2.7E-04 | 4.5E-04 | 1.5E-01 |
| 5 | 1.4E-09 | 2.7E-03 | 1.5E-02 | 2.1E-01 |
| 6 | 9.0E-05 | 1.6E-02 | 1.5E-01 | 2.1E-01 |
| 7 | 7.6E-02 | 6.4E-02 | 4.2E-01 | 1.6E-01 |
| 8 | 8.3E-01 | 1.6E-01 | 3.4E-01 | 9.1E-02 |
| 9 | 1.2E-01 | 2.5E-01 | 7.9E-02 | 3.7E-02 |
| 10 | 2.1E-04 | 2.5E-01 | 5.4E-03 | 1.1E-02 |
| 11 | 5.0E-09 | 1.6E-01 | 1.1E-04 | 2.5E-03 |
| 12 | 1.5E-15 | 6.6E-02 | 6.0E-07 | 4.1E-04 |
| 13 | 5.9E-24 | 1.7E-02 | 9.8E-10 | 4.9E-05 |
| 14 | 3.0E-34 | 2.8E-03 | 4.6E-13 | 4.4E-06 |
| 15 | 2.0E-46 | 3.0E-04 | 6.4E-17 | 2.9E-07 |
| **SUM** | **1** | **1** | **1** | **1** |
| **Mean+SD** | 8.1±0.2 | 9.5±0.4 | 7.3±0.4 | 5.6±1.0 |
| **n** | 163 | 93 | 395 | 46 |
| **P** | - | 2.0E-05^***^ | 8.8E-08^***^ | 8.7E-04^***^ |
| **Genotype** | ***Control***  *OregonR x w^1118^* | ***nos>Dl***  *nos-Gal4/*  *UASp-Dl* | ***nos>Dl^RNAi^***  *nos-Gal4/+; UASt-Dl^RNAi^* | ***germlineless***  ***mutant***  *tud^B42^* |

n – number of analyzed TFs

Kruskal-Wallis test was used to quantify the statistical significance

*P ≤ 0.05, **P ≤ 0.01, ***P ≤ 0.001

**S1B Table. Notch signaling controls the number of Terminal Filament Cells per individual Terminal Filament (TFCs/TF)**

| Possible TFCs/TF  numbers | Probability of various TFCs/TF numbers  based on the observations in different mutants | | | | | |
| --- | --- | --- | --- | --- | --- | --- |
| 1 | 3.1E-34 | 3.7E-04 | 1.3E-58 | 3.4E-12 | 1.5E-02 | 2.9E-03 |
| 2 | 3.1E-25 | 2.6E-03 | 9.5E-44 | 1.8E-09 | 4.4E-02 | 6.8E-03 |
| 3 | 1.2E-17 | 1.3E-02 | 4.3E-31 | 3.8E-07 | 9.7E-02 | 1.4E-02 |
| 4 | 1.8E-11 | 4.6E-02 | 1.2E-20 | 3.4E-05 | 1.6E-01 | 2.7E-02 |
| 5 | 1.1E-06 | 1.1E-01 | 2.1E-12 | 1.3E-03 | 2.1E-01 | 4.5E-02 |
| 6 | 2.4E-03 | 2.0E-01 | 2.4E-06 | 1.9E-02 | 2.0E-01 | 6.8E-02 |
| 7 | 2.1E-01 | 2.4E-01 | 1.7E-02 | 1.2E-01 | 1.4E-01 | 9.3E-02 |
| 8 | 7.0E-01 | 2.0E-01 | 7.7E-01 | 3.2E-01 | 8.0E-02 | 1.2E-01 |
| 9 | 9.1E-02 | 1.2E-01 | 2.2E-01 | 3.5E-01 | 3.4E-02 | 1.3E-01 |
| 10 | 4.6E-04 | 5.0E-02 | 3.8E-04 | 1.6E-01 | 1.1E-02 | 1.3E-01 |
| 11 | 9.0E-08 | 1.5E-02 | 4.3E-09 | 2.9E-02 | 2.6E-03 | 1.1E-01 |
| 12 | 6.8E-13 | 3.0E-03 | 3.0E-16 | 2.2E-03 | 4.7E-04 | 9.3E-02 |
| 13 | 2.0E-19 | 4.4E-04 | 1.3E-25 | 6.9E-05 | 6.5E-05 | 6.8E-02 |
| 14 | 2.2E-27 | 4.4E-05 | 3.8E-37 | 9.0E-07 | 6.9E-06 | 4.4E-02 |
| 15 | 9.8E-37 | 3.1E-06 | 6.7E-51 | 4.8E-09 | 5.5E-07 | 2.6E-02 |
| SUM | **1** | **1** | **1** | **1** | **1** | **1** |
| Mean+SD | 7.9±0.2 | 7.0±0.8 | 8.2±0.1 | 8.6±0.2 | 5.4±1.3 | 9.5±1.8 |
| n | 103 | 229 | 42 | 81 | 91 | 47 |
| P | **-** | **^a^ 1.5E-02^*^** | **^b^ 5.8E-01^n.s.^** | **^c^ 2.7E-03^**^** | **^c^ 3.4E-03^**^** | **^c^ 3.7E-02^*^** |
| Genotype | ***Control***  *N^ts1^*  kept at 18°C | ***Notch***  ***mutant***  *N^ts1^*  kept at 25°C | ***Control***  ***bab1>GFP***  *UASt-GFP/*  *bab1-Gal4* | ***bab1>N^CA^***  *UASt-N^CA^/*  *bab1-Gal4* | ***bab1>N^RNAi^***  *UASt-N^RNAi^/*  *bab1-Gal4* | ***bab1>Dl***  *UASt-Dl/*  *bab1-Gal4* |

n – number of analyzed TFs

Kruskal-Wallis test was used to quantify the statistical significance

^a^ - compared to *Control* (*N^ts^* kept at 18°C)

^b^ - compared to *Control* (*OregonR x w^1118^*)

^c^ - compared to *Control* (*bab1>UAS-GFP*)

*P ≤ 0.05, **P ≤ 0.01, ***P ≤ 0.001, n.s. - not significant

**S1C Table. Actin dynamics control the number of Terminal Filament Cells per individual Terminal Filament (TFCs/TF)**

| Possible TFCs/TF  numbers | Probability of various TFCs/TF numbers  based on the observations in different mutants | | | | | | | | |
| --- | --- | --- | --- | --- | --- | --- | --- | --- | --- |
| 1 | 1.6E-47 | 1.4E-12 | 1.8E-08 | 3.0E-09 | 1.1E-06 | 4.5E-11 | 1.1E-63 | 1.1E-06 | 1.7E-49 |
| 2 | 1.4E-35 | 2.4E-09 | 4.0E-06 | 6.4E-07 | 4.3E-05 | 3.4E-08 | 4.8E-47 | 4.3E-05 | 4.4E-36 |
| 3 | 2.0E-25 | 1.2E-06 | 3.1E-04 | 5.5E-05 | 9.3E-04 | 8.2E-06 | 6.0E-33 | 9.3E-04 | 7.9E-25 |
| 4 | 5.3E-17 | 1.8E-04 | 8.7E-03 | 1.9E-03 | 1.0E-02 | 6.5E-04 | 2.1E-21 | 1.0E-02 | 1.0E-15 |
| 5 | 2.4E-10 | 7.7E-03 | 8.7E-02 | 2.7E-02 | 6.1E-02 | 1.7E-02 | 2.1E-12 | 6.1E-02 | 8.9E-09 |
| 6 | 2.0E-05 | 9.7E-02 | 3.1E-01 | 1.5E-01 | 1.9E-01 | 1.4E-01 | 5.7E-06 | 1.9E-01 | 5.6E-04 |
| 7 | 2.8E-02 | 3.6E-01 | 3.9E-01 | 3.5E-01 | 3.1E-01 | 3.9E-01 | 4.4E-02 | 3.1E-01 | 2.4E-01 |
| 8 | 6.8E-01 | 3.9E-01 | 1.8E-01 | 3.2E-01 | 2.7E-01 | 3.4E-01 | 9.7E-01 | 2.7E-01 | 7.4E-01 |
| 9 | 2.9E-01 | 1.3E-01 | 2.8E-02 | 1.2E-01 | 1.2E-01 | 1.0E-01 | 5.9E-02 | 1.2E-01 | 1.6E-02 |
| 10 | 2.2E-03 | 1.2E-02 | 1.6E-03 | 1.8E-02 | 2.9E-02 | 9.5E-03 | 1.0E-05 | 2.9E-02 | 2.3E-06 |
| 11 | 2.9E-07 | 3.3E-04 | 3.3E-05 | 1.1E-03 | 3.7E-03 | 2.9E-04 | 4.8E-12 | 3.7E-03 | 2.4E-12 |
| 12 | 6.6E-13 | 2.7E-06 | 2.4E-07 | 2.7E-05 | 2.4E-04 | 2.9E-06 | 6.4E-21 | 2.4E-04 | 1.8E-20 |
| 13 | 2.6E-20 | 6.4E-09 | 6.3E-10 | 2.6E-07 | 8.4E-06 | 9.6E-09 | 2.4E-32 | 8.4E-06 | 9.0E-31 |
| 14 | 1.8E-29 | 4.5E-12 | 5.8E-13 | 1.0E-09 | 1.5E-07 | 1.0E-11 | 2.5E-46 | 1.5E-07 | 3.2E-43 |
| 15 | 2.3E-40 | 9.2E-16 | 1.9E-16 | 1.7E-12 | 1.5E-09 | 3.5E-15 | 7.5E-63 | 1.5E-09 | 7.9E-58 |
| SUM | **1** | **1** | **1** | **1** | **1** | **1** | **1** | **1** | **1** |
| Mean+SD | 8.0±0.1 | 7.5±0.5 | 6.7±0.5 | 7.4±0.6 | 7.2±0.6 | 7.4±0.5 | 8.0±0.1 | 7.4±0.7 | 7.7±0.1 |
| n | 57 | 131 | 22 | 148 | 101 | 118 | 42 | 101 | 116 |
| P | **^a^ 3.2E-01^n.s.^** | **^b^ 9.0E-03^**^** | **^b^ 4.5E-03^**^** | **^b^ 1.3E-02^*^** | **^b^ 3.3E-03^**^** | **^b^ 1.7E-03^**^** | **^b^ 3.6E-02^n.s.^** | **^c^ 1.2E-03**** | **^c^ 2.2E-03**** |
| Genotype | ***Control***  ***OregonR*** | ***Arp1^c^/+*** | ***SCAR^d37^/+*** | ***dia^5^/+*** | ***nos>***  ***SCAR^RNAi^*** | ***nos>***  ***Arp1^RNAi^*** | ***Control (bab1/+)*** | ***bab1> SCAR^RNAi^*** | ***bab1> Arp1^RNAi^*** |

n – number of analyzed TFs

Kruskal-Wallis test was used to quantify the statistical significance

^a^ - compared to *Control* (*OregonR x w^1118^*)

^b^ - compared to *Control* (*OregonR*)

^c^ - compared to *Control* (*bab1-Gal4/+*)

*P ≤ 0.05, **P ≤ 0.01, ***P ≤ 0.001, n.s. - not significant
